# Diversity of Flowering Responses in Wild *Arabidopsis thaliana* Strains

**DOI:** 10.1371/journal.pgen.0010006

**Published:** 2005-07-25

**Authors:** Janne Lempe, Sureshkumar Balasubramanian, Sridevi Sureshkumar, Anandita Singh, Markus Schmid, Detlef Weigel

**Affiliations:** 1 Department of Molecular Biology, Max Planck Institute for Developmental Biology, Tübingen, Germany; 2 Plant Biology Laboratory, Salk Institute for Biological Studies, La Jolla, California, United States of America; University of Wisconsin, United States of America

## Abstract

Although multiple environmental cues regulate the transition to flowering in *Arabidopsis thaliana*, previous studies have suggested that wild *A. thaliana* accessions fall primarily into two classes, distinguished by their requirement for vernalization (extended winter-like temperatures), which enables rapid flowering under long days. Much of the difference in vernalization response is apparently due to variation at two epistatically acting loci, *FRI* and *FLC*. We present the response of over 150 wild accessions to three different environmental variables. In long days, *FLC* is among those genes whose expression is most highly correlated with flowering. In short days, *FRI* and *FLC* are less important, although their contribution is still significant. In addition, there is considerable variation not only in vernalization response, but also in the response to differences in day length or ambient growth temperature. The identification of accessions that flower relatively early or late in specific environments suggests that many of the flowering-time pathways identified by mutagenesis, such as those that respond to day length, contribute to flowering-time variation in the wild. In contrast to differences in vernalization requirement, which are mainly mediated by *FRI* and *FLC*, it seems that variation in these other pathways is due to allelic effects at several different loci.

## Synopsis

Flowering is a quintessential adaptive trait in plants: Its correct timing ensures, for example, that plants do not produce seeds when they will not find favorable conditions for dispersal or germination. Befitting its importance, flowering is affected by several different environmental variables. The authors have compared the flowering times of over 150 *Arabidopsis thaliana* wild strains in response to three environmental factors: ambient growth temperature, day length, and vernalization (extended winter-like temperatures). Genetic and molecular analyses confirmed the important role of the previously identified *FRI* and *FLC* genes in flowering time. Genome-wide expression studies showed that *FLC* is among the genes whose expression is most highly correlated with flowering. Their studies, however, also revealed that the impact of *FRI* and *FLC* depends not only on vernalization treatment, which leads to repression of *FRI* and *FLC* activity, but also on day length. Within the groups of relatively early- and late-flowering strains, they find several unique responses, suggesting that many of the signaling pathways identified in mutant studies of laboratory strains are also being used to generate flowering diversity in the wild.

## Introduction

*Arabidopsis thaliana,* a facultative long-day plant, occurs throughout the northern hemisphere, and wild accessions show extensive variation in several traits including flowering time [1]. The major environmental factors that control flowering time are light quantity (day length) and quality, ambient growth temperature, and vernalization. In general, long days, high ambient temperature, and vernalization promote flowering, and many genes with positive and negative roles in mediating these effects have been identified, primarily through the analysis of laboratory-induced mutations.

Analysis of wild accessions has shown that there are winter annuals, in which flowering is strongly delayed unless plants are vernalized, as well as rapidly cycling strains [2,3]. A major factor that prevents *A. thaliana* from flowering rapidly without vernalization is *FRI (FRIGIDA)* [4]. *FRI* delays flowering by maintaining high expression levels of *FLC (FLOWERING LOCUS C)*, which encodes a MADS domain protein that represses flowering [5–7]. The widely used laboratory strains Landsberg *erecta* (L*er*) and Columbia (Col-0) lack *FRI* activity because of deletions at the *FRI* locus. These two deletions are found in many other rapid-cycling accessions, indicating that *FRI* is a major determinant of natural variation in flowering time [4]. In addition, less commonly occurring deletions and point mutations in *FRI* have been reported [8–10]. Apart from loss of *FRI* function, attenuation of *FLC* activity provides an alternative mechanism to achieve rapid-cycling behavior in wild strains [8,11].

Natural variation in the flowering responses to other environmental variables has not been studied in as much detail as the vernalization response has been analyzed. A naturally occurring deletion in *FLM*, which causes early flowering in both long and short days [12], has so far only been found in accessions from Niederzenz [13]. Similarly, the *EDI (EARLY DAY-LENGTH INSENSITIVE)* allele of the photoreceptor *CRY2 (CRYPTOCHROME 2)* causes early flowering under short days, but appears to be restricted to accessions from the Cape Verde Islands [14]. It has been proposed that other *CRY2* alleles affect flowering in a wide range of accessions, but experimental data supporting this suggestion are still lacking [15].

Despite the immense interest in exploiting natural variation to identify *A. thaliana* genes controlling flowering time, large studies that simultaneously compare the responses of wild accessions to different environments have been lacking. In three previous reports, summary statistics for short and long days, along with flowering times for about 70 strains in common garden experiments, were published [10,15,16]. In these 70 strains, *FRI* and *FLC* significantly delayed flowering only in long days. Here, we present detailed data on the flowering responses of over 150 wild strains along with several mutants under four different conditions. We report substantial variation in pathways other than those affected by vernalization, including the pathway that mediates responses to ambient temperature, which so far has not been studied in a range of natural accessions*.*

## Results/Discussion

### Extensive Variation in Flowering Responses of *A. thaliana* Accessions

We analyzed flowering time in a random set of accessions of single-seed descent, reflecting much of the geographic diversity represented in the European Arabidopsis Stock Centre (http://www.arabidopsis.info) at the time (Dataset S1; Table S1; Figure S1). We measured several traits, including days to flowering (DTF) and total leaf number (TLN), which were highly correlated under different conditions (*r* ^2^ = 0.6 to 0.9), with environment-dependent variation in growth rate found in only a few accessions. Heritability of TLN was higher than that of DTF (Table S2). We studied the effects of vernalization, ambient growth temperature, and photoperiod, using four different environments: 23 °C long days (23LD), 16 °C long days (16LD), 16 °C long days after 5 wk vernalization (16LDV), and 23 °C short days (23SD). DTF and TLN data from 155 wild strains, along with the common laboratory strains Col-0 and L*er* and 32 flowering time mutants in Col-0 or L*er* backgrounds, passed our quality controls in at least two conditions. A complete dataset across all environments was obtained from 177 strains.

**Figure 1:**
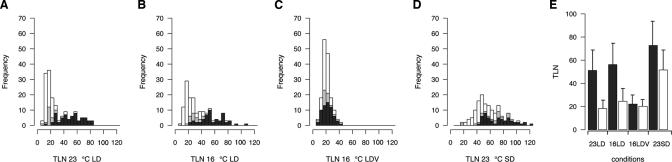
Distribution of Flowering Times among Wild Accessions (A–D) Distribution of flowering times expressed as TLN in23LD, 16LD, 16LDV, and 23SD. White and black bars represent accessions with and without Col- or L*er*-type deletions in *FRI*, respectively. Gray bars indicate strains subsequently identified to have defects in *FRI* or *FLC.* (E) Mean flowering times of putatively *FRI*/*FLC* functional (black bars) and nonfunctional (white bars) accessions in four environments.

In general, the distribution of flowering times reflected the action of genetic pathways known from the analysis of laboratory strains (Figure 1). In these, both long days and elevated temperatures accelerate flowering, and the wild accessions flowered on average earlier in 23LD compared to 23SD and 16LD. Similarly, a large tail of late-flowering accessions seen in 16LD largely disappeared upon vernalization. In contrast to the prominent peak of early-flowering accessions observed in 23LD (Figure 1A), flowering time in 23SD was more evenly distributed (Figure 1D).

**Figure 2:**
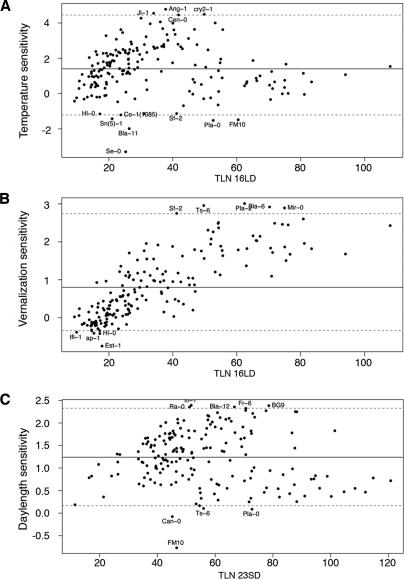
Environmental Responses Plotted against TLN Environmental responses were calculated from regression of TLN onto environmental means. (A) 23LD vs. 16LD; (B) 16LD vs. 16LDV; (C) 23LD vs. 23 SD; 95% of all values are between the gray dotted lines. The means are not centered because the responses are not normally distributed.

Having found substantial variation in the flowering time of accessions, we asked how similar the accessions were in their responses to environmental cues. The degree of genetic correlation between different environments indicated that the largest differences were in the response to vernalization, and the smallest in the response to ambient temperature (Table 1). Genotype-by-environment interactions accounted for 27% of flowering-time variation in response to vernalization (16LDV vs. 16LD); 9% in response to photoperiod (23LD vs. 23SD); and 3% in response to ambient temperature (23LD vs. 16LD), all of which were significant at *p* < 0.0001. The variation in sensitivity to different environmental factors was obvious when flowering times were regressed on the environmental mean (Figure 2) [17]. Accessions also differed extensively in other traits related to flowering, such as juvenile, adult, and cauline leaf number, indicating further differences in developmental physiology (see Table S1). Taken together, these results demonstrate that there is extensive variation in the responses of accessions to several environmental cues.

**Table 1:**
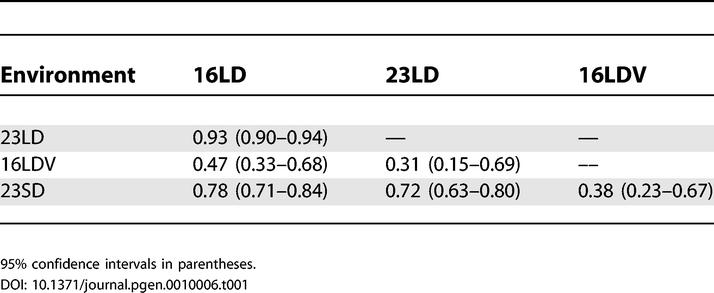
Genetic Correlation across Environments

95% confidence intervals in parentheses.

Co-variation between environmental factors and a particular trait can be evidence for selection. Environmental factors such as light and temperature, in turn, are strongly correlated with latitude, and latitudinal clines for different phenotypes are not uncommon [18]. Previously, a strong correlation between flowering time and latitude was demonstrated for accessions that were sown in fall and overwintered in a common garden in Rhode Island. Among strains with apparently functional *FRI*/*FLC* (see below), this correlation was particularly high (*r* ^2^ = 0.38, *n* = 21) [10].

**Figure 3:**
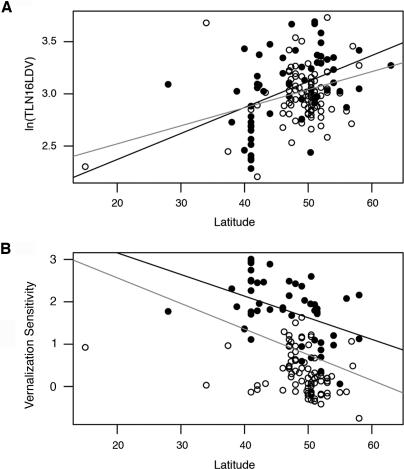
A Latitudinal Cline in Flowering after Vernalization Correlation of latitude with (A) flowering time, (B) and vernalization sensitivity (expressed as the regression coefficient of TLN on 16LD and 16LDV grand means). Both accessions with putatively functional alleles (black dots) and nonfunctional alleles (grey dots) at *FRI/FLC* are shown. Regression line with only *FRI/FLC* functional accessions (black line) and with all accessions (grey line) is shown separately. *FRI*/*FLC* functional accessions are indicated by black dots, *FRI*/*FLC* nonfunctional accessions by hollow circles. The correlation of flowering in *FRI*/*FLC* functional accessions with latitude (black line; *r* ^2^ = 0.18) is higher than of all accessions (grey line; *r* ^2^ = 0.13).

In our set of accessions, we found the strongest correlation between latitude and flowering (TLN) in 16LDV (Figure 3A; *r* ^2^ = 0.18, *p* < 0.0001), suggesting that the latitude correlation in the aforementioned common garden experiment is dependent on vernalization during natural winter conditions. This correlation is even higher in the group of accessions that was shared between our study and that of Stinchcombe and colleagues [10], namely *r* ^2^ = 0.38, *n* = 9. Thus, the differences in the strength of the latitudinal cline that we and Stinchcombe and colleagues [10] observed might be due to different sampling biases.

Consistent with flowering time being correlated with the magnitude of the vernalization response, there is also a significant latitudinal cline in the vernalization sensitivity of accessions (Figure 3B; *r^2^* = 0.12, *p* < 0.0001). Because there was very little correlation between latitude and longitude for the origin of strains (*r* ^2^ = 0.035), we did not include longitude as a covariant in our analyses. Although population structure may be a confounding factor in evaluating latitudinal clines, this concern is somewhat mitigated by the relatively small amount of population structure in *A. thaliana* [19,20].

### The Contribution of *FLC* Activity to Flowering-Time Variation

**Figure 4:**
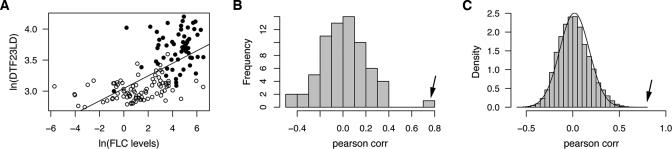
Correlation of *FLC* Expression with Flowering Time (A) Correlation of *FLC* RNA levels and DTF in 149 accessions, as measured by qRT-PCR. Hollow circles indicate accessions with lesions in *FRI.* (B) Correlation of expression levels of 68 flowering regulators with flowering time. Arrow indicates *FLC*. (C) Correlation of expression estimates (expressed as log2) of 22,500 genes represented on Affymetrix ATH1 array with flowering times across 34 accessions. Arrow indicates *FLC*, and line indicates distribution from 1,000 permutations.

In both laboratory-induced mutant strains and natural accessions, there is broad correlation of *FLC* expression levels, as determined by RNA blots, and flowering time, such that plants flower late when *FLC* levels are high and early when *FLC* levels are low [5,6,8,11,21]. However, a recent study of 17 accessions revealed also considerable variation in *FLC* expression among late-flowering accessions [22]. To evaluate more precisely the relationship between absolute *FLC* levels and quantitative variation in flowering time, we analyzed *FLC* expression by qRT-PCR in 149 accessions grown at 23LD (Figure 4A). Across all strains, variation in *FLC* levels accounted for 42% and 40% of TLN and DTF variation (*r* ^2^), respectively (*p* < 0.0001). As expected, this is largely due to *FLC* mediating the vernalization response, which can be calculated as the relative reduction in TLN or DTF when 16LDV and 16LD are compared. The correlation of *FLC* expression level with vernalization response using TLN and DTF is 47% and 52%, respectively.

In other members of the Brassicaceae family, loci controlling flowering time co-localize with *FLC* orthologs [23], but the relationship between expression of different *FLC* paralogs and flowering time appears to be more complex than in *A. thaliana* [24,25]. Furthermore, *FLC* expression in *Brassica oleracea* var. *capitata* shows differential responses to vernalization, depending on the tissue examined [26].

To determine whether the substantial correlation between *FLC* expression levels and flowering time across 149 *A. thaliana* accessions was unusual compared to other genes known to control flowering, or even to the remainder of the transcriptome in general, we examined Affymetrix ATH1 array data for 34 accessions, which had been generated as part of the AtGenExpress project (Table S3). The correlation of *FLC* with TLN and DTF at 23LD that we obtained using array analyses was 37% and 42%, respectively, which is quite similar to the 42% and 40% estimated from qRT-PCR analyses. We found that, among a set of 68 floral regulators, *FLC* was the one most highly associated with either TLN or DTF (Figure 4B; Table S4). In addition, when examining other conditions, we found none of the other floral regulators were as highly correlated with flowering time as *FLC* was for 23LD.

When we considered all genes represented on the ATH1 array, we found *FLC* to be among the ten most highly correlated genes at 23LD (both positively and negatively correlated) (Figure 4C). This was true regardless of whether expression was regressed linearly or logarithmically onto flowering time, and whether Pearson or Spearman rank correlation was calculated. With respect to DTF, *FLC* levels were considerably more correlated with this trait than expression levels of any other gene. We determined significance by permutation analysis, and found that *FLC* was the only gene significantly correlated with DTF at a *p* < 0.05 level under both Pearson and Spearman rank correlation. The prominence of *FLC*, whose levels are highly correlated with vernalization response, may reflect the fact that this response is the most important variable affecting flowering time in *A. thaliana* accessions. We note, however, that the analysis was biased, because we analyzed young seedlings, a stage in which *FLC* levels are particularly high.

**Figure 5:**
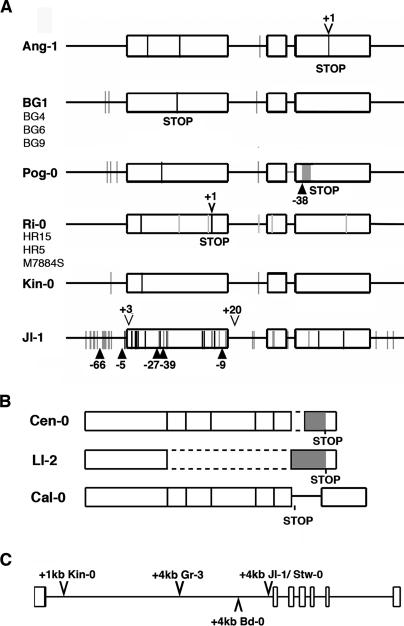
Newly Identified Alleles of *FRI* and *FLC* (A) Diagrams of *FRI* alleles. Exons are represented by boxes, introns by lines. Black lines indicate nonsynonymous changes, gray lines synonymous changes as well as polymorphisms in the non-coding region compared to the reference sequence of H51 [4]. Insertions and deletions (indels) are designated by triangles, numbers indicate size in base pairs (bp). Premature stop codons are caused by a 1-bp insertion in Ang-1, single bp polymorphisms in BG1 and Ri-0, or a deletion and inversion (gray box) in Pog-0. Note extensive polymorphisms in Jl-1. BG4, BG6, BG9, and HR5 were only partially sequenced. (B) Changes in *FLC* transcripts in three accessions. Dotted lines indicate exons that are missing in part or completely. These deletions cause frame shifts and thus altered amino acid sequences (grey boxes). Premature stop codons are indicated. The reasons for the aberrant transcript processing in Ll-2 are not known. In Cen-0, an alternative splice acceptor site in the last exon is used, leading to a deletion of exon sequences and a frame shift, whereas in Cal-0 an alternative splice acceptor site in the last intron is used, which adds additional sequences and also causes a frame shift of sequences of the last exon. (C) Large insertions in the first intron of *FLC* in several accessions.

There was a general association of low *FLC* levels with the Col- and L*er*-type deletions in *FRI* and early flowering, but this relationship was not absolute. Several exceptions to this rule are explained by other nonfunctional *FRI* alleles or variation in *FLC* itself, as shown by a combination of genetic and molecular analyses (Table 2; Figure 5; Figure S2). Several of the early accessions have missense mutations in *FRI* that are different from those described before [8,9], whereas one nonfunctional *FRI* allele has extensive polymorphisms (Figure 5A). Because the effects of *FRI* on flowering time are entirely dependent on *FLC* [7], we were also curious whether there are any molecular effects of *FRI* in the absence of *FLC*. To this end, we compared the global expression profile at the shoot apex in response to floral induction by long days between *FRI flc*-3 and *fri*-Col *flc*-3 plants, similar to a design used before [27]. We detected no significant differences between the two genotypes (data not shown), confirming that *FRI* function absolutely requires *FLC* activity.

**Table 2:**
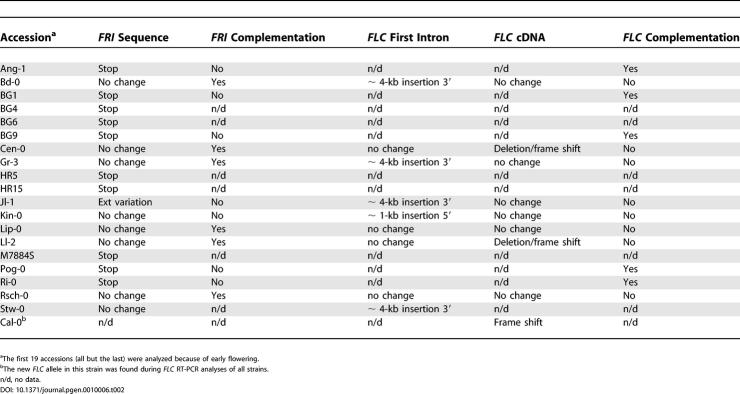
Accessions with Compromised *FRI* or *FLC* Activity

^a^The first 19 accessions (all but the last) were analyzed because of early flowering.

^b^The new *FLC* allele in this strain was found during *FLC* RT-PCR analyses of all strains.

n/d, no data.

Although null alleles of *FRI* are easily found in natural accessions [4,9], only transposon insertions with reduced expression level of a wild-type transcript have been described for *FLC* [6,8,11,16], which has led to the proposal that null alleles of *FLC* have reduced fitness in the wild [3]*.* However, we discovered three natural *FLC* alleles with severely affected protein function (Figure 5B). At least one of the very early accessions, Ll-2, likely carries a null allele, because transgenic overexpression of the Ll-2 *FLC* cDNA, which lacks exons 2 to 6, in an *flc*-3 background has no effect on flowering (not shown). These findings demonstrate that *FLC* alleles with severely compromised protein function can be recovered from wild populations. We also found several new transposon insertions in the first intron of *FLC* (Figure 5C).

### Variation in Responses to Photoperiod and Ambient Temperature

In total, our collection of 155 strains included 67 strains that carry either the common Col- or L*er*-type lesions in *FRI*, and at least 23 accessions with alternative *FRI/FLC* lesions. A two-way analysis of variance (ANOVA) model showed that *FRI* and *FLC* can account for 63% of variation in 23LD TLN. In contrast, *FRI* and *FLC* accounted for only 23% variation in 23SD, consistent with this pathway being most important when other floral inductive stimuli are strong, as in long days. Nevertheless, our finding that *FRI* and *FLC* significantly affect flowering in short days in a large set of wild strains is at variance with previous surveys that did not find a significant effect of *FRI* [10,16]. Twenty-four wild strains are in common between the previous studies and our study. A comparison of flowering times indicates limited correlation in these 24 strains (*r* ^2^ = 0.55 for long days, 0.35 for short days). Consistent with the differences in observed flowering time, *FRI* and *FLC* are significant factors in our long day and short day conditions in this set of 24 strains, indicating that these discrepancies are not due to sampling bias, but different experimental conditions. Our results are consistent with earlier studies using *FRI/FLC* introgressed laboratory strains, which also displayed late flowering in short days [28,29]. In addition, we did not limit our assessment of *FRI* and *FLC* activity to genotyping of major, previously known alleles.

It has been suggested that a disadvantage of growth chamber experiments compared to common garden experiments is an increased contribution of random environmental variation, which may obscure ecologically relevant responses [10,30]. We believe that we have been able to limit random environmental variation with very accurate growth facilities along with a completely randomized experimental design (see Materials and Methods).

Consistent with *FRI* and *FLC* mediating most of the vernalization response, there is no significant difference between groups with and without *FRI*/*FLC* activity in 16LDV, suggesting that our vernalization treatment is largely saturating (see Figure 1E). However, although vernalization strongly reduces late flowering, it does not abolish variation in flowering time, which still varies more than 4-fold (see Figure 1C).

**Figure 6:**
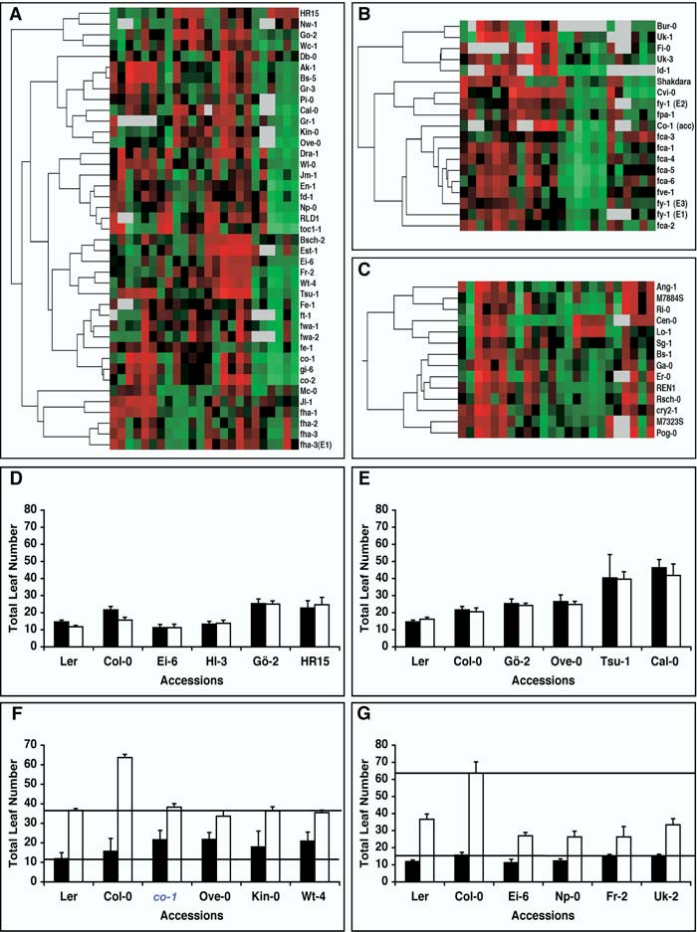
Variation in Responses to Environmental Cues Other than Vernalization (A–C) Hierarchical clustering identifies groups with different flowering behaviors (see Figure S3 for entire cluster diagram). Green and red indicate earlier and later flowering than the mean, respectively. Gray indicates missing data. Conditions are, from left to right: 16LD, 23LD, 16LDV, 23SD; with six columns each (DTF, juvenile rosette leaf number, adult rosette leaf number, rosette leaf number, cauline leaf number, TLN). (A) Accessions that cluster with photoperiodic mutants. (B) Accessions that cluster with mutants of the autonomous pathway. (C) Accessions that flower late in 16LD. (D–G) Comparison of specific accessions with laboratory strains Col-0 and L*er*. (D) Accessions that are temperature-insensitive and not delayed in 16LD (black) compared to 23LD (white bars). (E) Accessions that do not respond to vernalization and flower similarly in 16LD (black) and 16LDV (white). (F) Accessions that flower late in long days (23LD, black), but not short days (23SD, white). For comparison, the photoperiodic *co-*1 mutant is included. (G) Accessions that flower early in short days (23SD, white) compared to long days (23LD, black).

One of our long-term goals is to identify genes and pathways that contribute to vernalization-independent variation in flowering time of natural accessions. To identify candidate accessions with interesting variation in flowering responses, we applied hierarchical clustering, which has been used before to group accessions according to light sensitivity during seedling development [31] (Figures 6A–6C, S3–S5). Potentially interesting accessions were also identified as those having large residuals when comparing contrasting conditions, such as 23LD versus 16LD, 23LD versus 23SD, and 16LD versus 16LDV. These specific comparisons yielded similar results to hierarchical clustering (Figure 6D–6G).

To associate accessions with variation in specific genetic pathways, the cluster analysis included a large number of known flowering-time mutants. The first major distinction was between accessions that flowered either relatively early or late in long days, with the first group comprising accessions that carry lesions at *FRI* or *FLC*. The second cluster consisted of late-flowering, vernalization-responsive accessions along with strains that behaved similarly to very late-flowering photoperiodic mutants such as *constans* (*co*) or *gigantea* (*gi*) [32,33]*.* Because all the mutants we examined had been isolated from backgrounds that lacked *FRI* function, we independently clustered rapid-cycling accessions with laboratory-induced mutants (see Figure S4), which resulted in several major groups, two of which were similar to photoperiodic mutants in that there was relatively little difference in flowering under long and short days. One was relatively late in long days (Figure 6F), whereas the other was relatively early in short days (Figure 6G). Another group behaved similarly to autonomous pathway mutants and included accessions that were rather late in 16LD and 23LD, but not in 23SD (Figure 6B).

We looked specifically for variation among putatively *FRI*/*FLC* functional accessions, by clustering them together with some of the accessions that we had identified as lacking *FRI*/*FLC* activity. This resulted in three major groups, which differ in their vernalization sensitivity (Figure S5). Several accessions from lower latitudes were found in a class that becomes particularly early after vernalization (Figure S5). This observation, although consistent both with our observation of a latitudinal cline in vernalization sensitivity (Figure 5B) and with earlier findings [10], does not imply that *FRI* promotes flowering after vernalization, but rather may reflect that other mechanisms, which otherwise delay flowering, are dispensable in this cluster of accessions.

**Figure 7:**
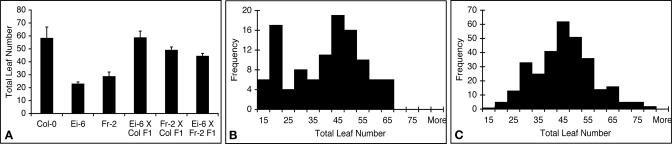
Genetic Analysis of Two Accessions that Flower Early in 23SD (A) Flowering time of accessions and F_1_ progeny. (B) A distinct class of early-flowering plants in the Fr 2 × Col-0 F_2_ population. (C) Continuous segregation of flowering times in Ei-6 × Col-0 F_2_ population.

As a first step toward identifying the genetic mechanisms underlying the newly identified flowering behaviors, we examined F_2_ populations from crosses of Ei-6 and Fr-2, two strains that flower relatively early in 23SD, to the reference strain Col-0. In both cases, the earliness appeared to be recessive compared to Col-0 (Figure 7A). Consistent with the recessive nature of earliness, neither strain carries the naturally occurring, dominantly acting *EDI* allele of *CRY2*, which can cause early flowering in short days accession [14]. In addition, when we crossed Ei-6 and Fr-2 to each other, the F_1_ hybrids were later than either parent, indicating that earliness in short days has a different genetic basis in these strains (Figure 7A). The F_2_ progeny from a cross of Fr-2 to Col-0 included a distinct early class, suggesting that early flowering is due to variation at a major locus (Figure 7B). In contrast, in the Ei-6 × Col-0 cross, continuous variation can be seen, indicating the involvement of multiple loci (Figure 7C).

### Conclusions

We provide an extensive dataset on flowering-time associated traits in a large number of *A. thaliana* accessions across multiple environments, which forms a valuable resource for both experimental and population genetic studies. We have tested the response to three major environmental variables affecting flowering time: photoperiod, vernalization, as well as ambient growth temperature, whose effects had not been studied before in natural strains. Although our data confirm that *FRI* and *FLC* are indeed major factors regulating the rapid-cycling or winter-annual habit, loss of *FRI* and *FLC* function cannot explain all of the variation observed. Some of the remaining variation may be due to more subtle differences in the activity of the vernalization pathway*.*

Although there is less variation in the ambient temperature response compared to vernalization and photoperiod, we could identify accessions that responded more strongly or more weakly to temperature than the majority of accessions (see Figure 6C and 6D). The identification of accessions that flower relatively early or late in specific environments suggests that many of the flowering-time pathways identified by mutagenesis, such as those that respond to photoperiod or temperature, participate in generating flowering-time variation in the wild. In contrast to variation in vernalization requirement, which appears to be largely due to *FRI* and *FLC*, it seems that variation in these other pathways is due to allelic effects at several different loci, a conclusion that is supported by a growing number of other studies [13,14,22]. The hypothesis of a complex genetic basis of non-vernalization variation is further supported by the finding that, in contrast to vernalization response, none of the known flowering-time regulators are particularly highly correlated with photoperiod or temperature response. It will be interesting to learn which genes control variation in these other pathways.

## Materials and Methods

### 

#### Stocks.

Accessions were obtained from the Nottingham Arabidopsis Stock Centre (http://www.arabidopsis.info). Strains with different combinations of *FRI* and *FLC* alleles have been described [6,7,28].

#### Growth conditions.

Plants were grown in controlled growth rooms with a temperature variability of about plus or minus 0.1 °C under a 1:1 mixture of Cool White and Gro-Lux Wide Spectrum fluorescent lights, with a fluence rate of 125 to 175 μmol m^−2^ s^−1^. All light bulbs were of the same age. Long days had 16 h of light, short days had 8 h. Maximal humidity was 65%. Light, temperature, and humidity were continuously monitored online and logged data were stored in a Structured Query Language (SQL) database.

#### Plant culture.

Seeds were stratified at 4 °C for 7 d (to minimize variation due to differences in stratification requirements) in 0.1% agarose, then planted on soil. Before vernalization, seed germination was induced at 16 °C for 24 h. Plants were cultivated for 5 wk at 4 °C in a vernalization room with 8 h light of about 50 μmol m^−2^ s^−1^, before transfer to normal growth rooms.

#### Experimental design.

Twelve seeds per genotype were sown in a completely randomized design in individually numbered positions of multiple-well flats. If more than one seed had been deposited at a certain position, only one plant was allowed to remain after 1 wk. Flats were watered on alternating days and rotated every time to minimize the effects of positional light or stagnant water. Only genotypes with at least four germinated plants in a given environment were included in further analysis. Typically, eight to ten plants per genotype and condition were analyzed. For the first set of experiments (which included mostly the vernalization-requiring accessions), seeds from the stock center were used. For the second set of experiments, progeny derived by single-seed descent were used. We analyzed the efficiency of randomization by observing the total average across shelves. There was no statistically significant variation across the shelves.

#### Scoring of traits.

The following traits were scored: DTF, TLN, juvenile leaf number (JLN), adult leaf number (ALN), cauline leaf number (CLN), and rosette leaf number (RLN). Flowering was scored on a daily basis for first macroscopic appearance of the inflorescence. DTF was calculated from the date of sowing. For vernalized plants, the vernalization period was subtracted. In our conditions, plants did not produce leaves during vernalization. Individual plants that had flowered were removed. Flats remained in their specific conditions throughout the entire experiment. Leaves were counted under the microscope, and juvenility was determined by the presence of trichomes on the abaxial side.

#### Quality control.

As a first step of quality control, we analyzed variation between identical genotypes that had been included in both sets of experiments. No statistically significant variation could be detected. Therefore, we pooled the two datasets and treated them as a single experiment for further analysis. Some accessions displayed unusually large variation, suggestive of segregation or seed contamination. We therefore removed those genotypes from the analysis.

#### Statistical analyses.

Data were analyzed using JMP (version 5.1, SAS Institute, Cary, North Carolina, United States), the “base” package of R (http://www.R-project.org) [34], or Excel (Microsoft, Redmond, Washington, United States). The total variance was partitioned into between-line variance and the residuals with a one-way ANOVA using phenotype as a response and accession as a single factor of random effect. The variance component was estimated using a restricted maximum likelihood method (REML). Broad-sense heritability (*H* ^2^) was calculated as between-line variance (*V*_G_) divided by total variance. The coefficient of genetic variation (*CV*_G_) was calculated as (100 × 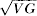)/mean for each trait. Genetic correlations (*r*_GE_) were calculated from covariance and variance components estimated through REML, using those genotypes for which data across all environments were available. The significance of each genetic correlation was determined using a *t*-test after *Z* transformation of the correlation coefficient [35]. Genotype-by-environment interactions were tested using a two-way ANOVA with strain and conditions as classifying factors. Hierarchical cluster analysis was performed with Cluster 3.0 [36]. The data were normalized to the overall mean, followed by log transformation. Noncentric, average linkage (UPGMA) clustering was then performed using Pearson correlation. To evaluate environmental sensitivity, TLN was regressed onto the environmental mean, [log(TLN_environmentA_) − log(TLN_environmentB_)]/[log(mean TLN_environmentA_) − log(mean TLN_environmentB_)] [17].

#### DNA analyses.

DNA was extracted using the CTAB method [27] with minor modifications. For sequence analysis of *FRI* and *FLC,* 3.3 kb and 4.2 kb genomic fragments, respectively, were amplified using ExTaq polymerase (TaKaRa Biomedical, Shiga, Japan). Pooled products from four independent PCR reactions were directly sequenced on both strands. See Table S5 for oligonucleotide primers used.

#### Expression studies.

The aerial parts of plants grown on soil in 23LD for 12 d were collected at dusk and flash frozen in liquid nitrogen. RNA was extracted using Trizol, and 1 μg of total RNA was reverse transcribed using a Reverse Transcription kit (Invitrogen, Carlsbad, California, United States). See Table S5 for a list of oligonucleotide primers. PCR was performed in the presence of SYBR Green (Invitrogen), and amplification was monitored in real time with the Opticon Continuous Fluorescence Detection System (MJ Research, Reno, Nevada, United States). Two biological replicates, each with two technical replicates, were analyzed. Threshold cycles (cT) were based on a reaction reaching a specific fluorescence intensity in the log-linear phase of the amplification curve. ΔcT was calculated as the difference in cT between *FLC* and *UBQ10*. PCR efficiency was assumed to be the same and relative transcript abundance compared to Col-0 was calculated as 2^−ΔΔcT^.

Microarray data were generated as part of the AtGenExpress project (http://arabidopsis.org/info/expression/ATGenExpress.jsp) (see Table S3). RNA isolated from the aerial parts of 4-d-old seedlings was hybridized to Affymetrix ATH1 arrays as described [27], and normalized expression estimates were obtained using gcRMA (bioconductor.org), a modification of the robust multi-array analysis (RMA) algorithm [37]. Microarray data have been deposited with the ArrayExpress database (http://www.ebi.ac.uk/arrayexpress; experiment E-TABM-18 and E-TABM-19). In addition, normalized microarray data are available from our website (http://www.weigelworld.org/resources/microarray).

## Supporting Information

Dataset S1 Means of Flowering Time Traits in Accessions and Mutant Strains Data file of flowering-time-related traits in accessions and mutants. Also available as a PDF file (Table S1).
(38 KB CSV)

Figure S1 Geographic Distribution of Wild Strains Used in the Survey Open circles indicate known *FRI*/*FLC* defects.
(47 KB PDF)

Figure S2 Genetic Analysis of Rapid-Cycling Accessions Flowering times of F_1_ progeny from crosses of accessions that do not carry L*er*- or Col-type deletions in *FRI* to *fri*-Col *flc*-3 (white), *FRI*-Sf2 *flc*-3 (gray), *fri*-Col *FLC* (black), and *FRI*-Sf2 *FLC* (striped).
(A) F_1_ progeny in which *FRI* causes late flowering.
(B) F_1_ progeny in which *FLC* causes late flowering.
(C) F_1_ progeny in which only simultaneous introduction of *FRI* and *FLC* causes late flowering.
(78 KB PDF)

Figure S3 Hierarchical Cluster Analysis of All Genotypes Columns for each condition are DTF, JLN, ALN, RLN, CLN, and TLN. Hierarchical clustering identifies groups with different flowering behaviors. Green indicates earlier flowering than the mean, red later flowering than the mean, and gray indicates missing data.
(110 KB PDF)

Figure S4 Hierarchical Clustering of Accessions Lacking Functional *FRI* or *FLC*
(A) Accessions that are similar to Col.
(B) Accessions that are similar to L*er*, with a reduced adult phase at 16LD.
(C) Accessions that are severely delayed in 16LD compared to 23LD.
(D) Accessions that cluster together with mutants of the photoperiodic pathway. This group includes several accessions that flower early in 23SD.
(E) Accessions that cluster with mutants of the autonomous pathway.
(950 KB PDF)

Figure S5 Hierarchical Clustering of Accessions with Functional *FRI/FLC* Pathway (Top) Accessions with moderate vernalization response.
(Middle) Accessions with extreme vernalization response. Several accessions in this cluster originate from lower latitudes (given in parentheses).
(Bottom) Accessions that are delayed after vernalization, possibly indicating *FRI*-independent variation. Note that BG6, HR5, and Wil-1, which lack functional *FRI*, cluster with this group.
(594 KB PDF)

Table S1 Means of Flowering Time Traits in Accessions and Mutant Strains A, B, C, etc., denote multiple stocks and repeated assays. Latitude information was obtained from Geographic Names Information System (US Geological Survey).
ALN, adult rosette leaf number; CLN, cauline leaf number; DTF, days to flowering; JLN, juvenile rosette leaf number; TLN, total leaf number.
The data are also available as a CSV file (Dataset S1).
(62 KB PDF)

Table S2 Summary Statistics Based on Individual Plants Asterisk indicates ± 95% confidence intervals (2 × SEM).
(31 KB PDF)

Table S3 Key to ATH1 Array Data (33 KB PDF)

Table S4 Pearson Correlation of Known Flowering Regulators with DTF and TLN in 23LD (33 KB PDF)

Table S5 Oligonucleotide Primers Used Uses: 1/3, amplification of 3.3-kb *FRI* genomic region; 1–13, *FRI* sequence analysis; 12/13, PCR genotyping of Col-type deletion in *FRI;* 3/4, PCR genotyping of L*er*-type deletion in *FRI;* 6/7, *FRI* qRT-PCR; 30/31 and 28/34, amplification of 4.2-kb *FLC* genomic region (divided into two fragments); 14–35, *FLC* sequence analysis; 34/35, *FLC* cDNA amplification; 36–38, PCR genotyping of L*er*-type insertion in *FLC;* 39/40, *UBQ10* qRT-PCR; 41/42, *FLC* qRT-PCR.
(36 KB PDF)

### Accession Numbers

GenBank (http://www.ncbi.nlm.nih.gov/Genbank/) accession numbers of the new sequences discussed in this paper are AY964090–AY964098 and AY0528–AY970557.

## Abbreviations

Col-0: Columbia
DTF: days to flowering
TLN;: total leaf number
L*er*: Landsberg *erecta*
16LD: 16 °C long days
16LDV: 16 °C long days after 5 wk vernalization
23LD: 23 °C long days
23SD: 23 °C short days
