# Supplementary material for: Diversity of Flowering Responses in Wild Arabidopsis thaliana Strains
Source: PLoS Genet. 2005 Jul 25;1(1):e6. doi: 10.1371/journal.pgen.0010006 (PMC1183525; doi:10.1371/journal.pgen.0010006)
Supplement: Figure S1 — Open circles indicate known FRI/FLC defects. (47 KB PDF) [file pgen.0010006.sg001.pdf]

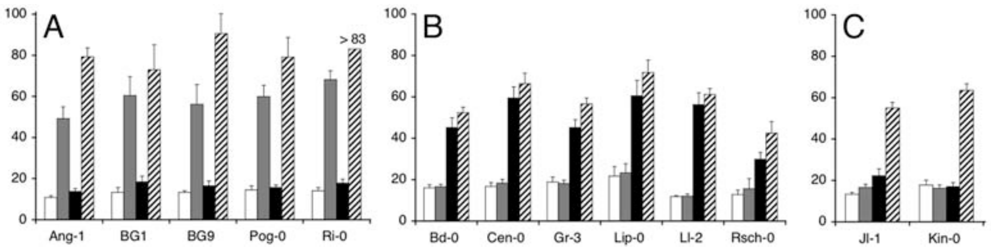

Supplementary Figure 2. Genetic analysis of rapid-cycling accessions.

Flowering times of F_1 progeny from crosses of accessions that do not carry L*er*- or Col-type deletions in *FRI* to *fri*-Col *flc*-3 (white), *FRI*-Sf2 *flc*-3 (gray), *fri*-Col *FLC* (black), *FRI*-Sf2 *FLC* (striped). (*A*) F_1 progeny in which *FRI* causes late flowering. (*B*) F_1 progeny in which *FLC* causes late flowering. (*C*) F_1 progeny in which only simultaneous introduction of *FRI* and *FLC* causes late flowering.
