# Supplementary material for: Diversity of Flowering Responses in Wild Arabidopsis thaliana Strains
Source: PLoS Genet. 2005 Jul 25;1(1):e6. doi: 10.1371/journal.pgen.0010006 (PMC1183525; doi:10.1371/journal.pgen.0010006)
Supplement: Figure S3 — Columns for each condition are DTF, JLN, ALN, RLN, CLN, and TLN. Hierarchical clustering identifies groups with different flowering behaviors. Green indicates earlier flowering than the mean, red later flowering than the mean, and gray indicates missing data. (110 KB PDF) [file pgen.0010006.sg003.pdf]

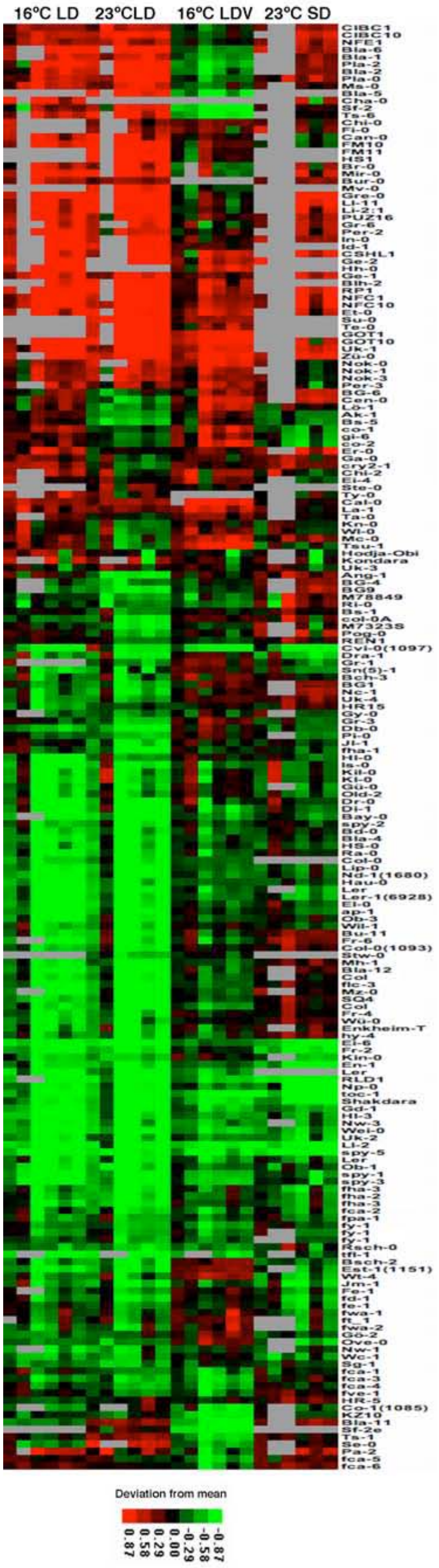

Supplementary Figure 3. Hierarchical cluster analysis of all genotypes.

Columns for each condition are DTF, JLN, ALN, RLN, CLN, TLN. Hierarchical clustering identifies groups with different flowering behaviors. Green indicates earlier flowering than the mean, red later flowering than the mean, and gray indicates missing data.
