# Supplementary material for: Diversity of Flowering Responses in Wild Arabidopsis thaliana Strains
Source: PLoS Genet. 2005 Jul 25;1(1):e6. doi: 10.1371/journal.pgen.0010006 (PMC1183525; doi:10.1371/journal.pgen.0010006)
Supplement: Figure S4 — (A) Accessions that are similar to Col. (B) Accessions that are similar to Ler, with a reduced adult phase at 16LD. (C) Accessions that are severely delayed in 16LD compared to 23LD. (D) Accessions that cluster together with mutants of the photoperiodic pathway. This group includes several accessions that flower early in 23SD. (E) Accessions that cluster with mutants of the autonomous pathway. (950 KB PDF) [file pgen.0010006.sg004.pdf]

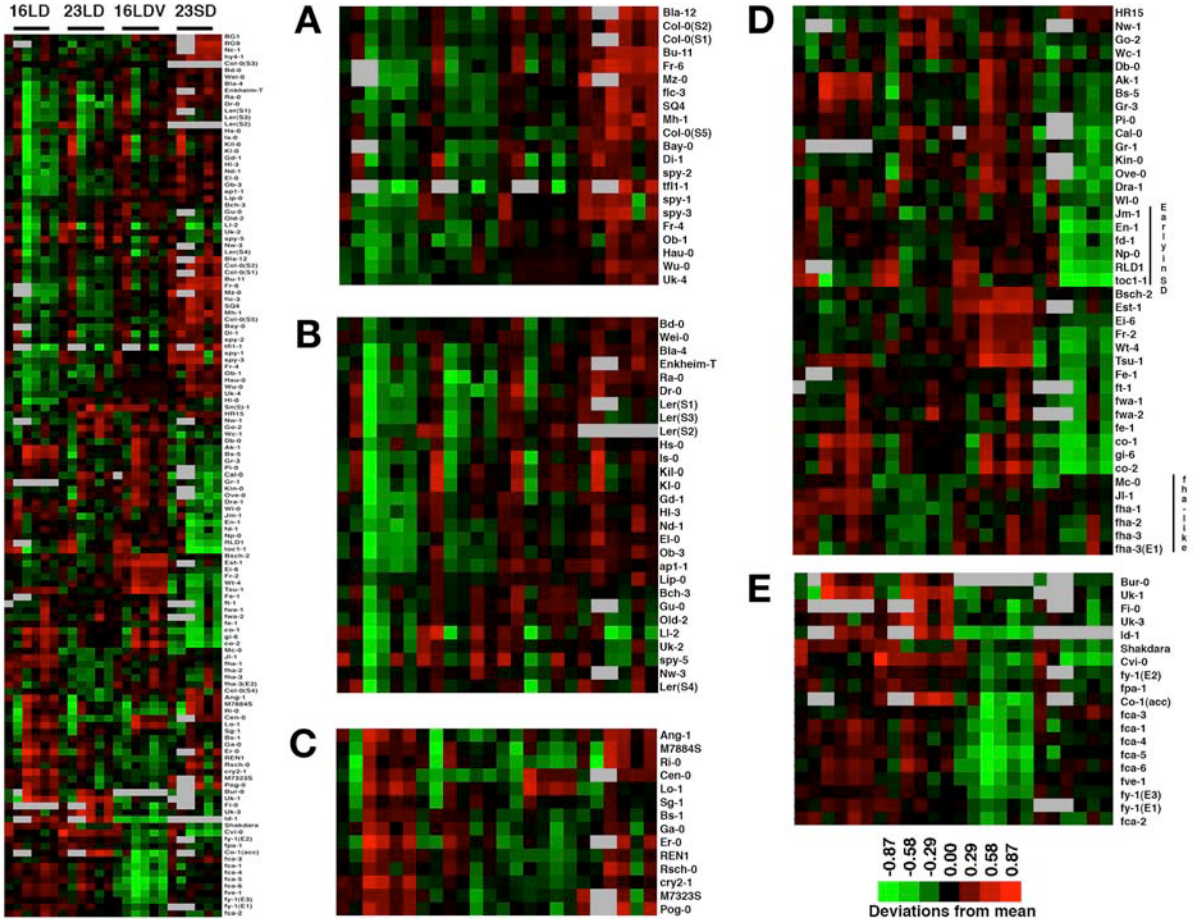

Supplementary Figure 4. Hierarchical clustering of accessions lacking functional *FRI* or *FLC.* (*A*) Accessions that are similar to Col. (*B*) Accessions that are similar to L*er*, with a reduced adult phase at 16°C LD. (*C*) Accessions that are severely delayed in 16LD compared to 23LD. (*D*) Accessions that cluster together with mutants of the photoperiodic pathway. This group includes several accessions that flower early in 23SD. (*E*) Accessions that cluster with mutants of the autonomous pathway.
