# Supplementary material for: Diversity of Flowering Responses in Wild Arabidopsis thaliana Strains
Source: PLoS Genet. 2005 Jul 25;1(1):e6. doi: 10.1371/journal.pgen.0010006 (PMC1183525; doi:10.1371/journal.pgen.0010006)
Supplement: Figure S5 — (Top) Accessions with moderate vernalization response. (Middle) Accessions with extreme vernalization response. Several accessions in this cluster originate from lower latitudes (given in parentheses). (Bottom) Accessions that are delayed after vernalization, possibly indicating FRI-independent variation. Note that BG6, HR5, and Wil-1, which lack functional FRI, cluster with this group. (594 KB PDF) [file pgen.0010006.sg005.pdf]

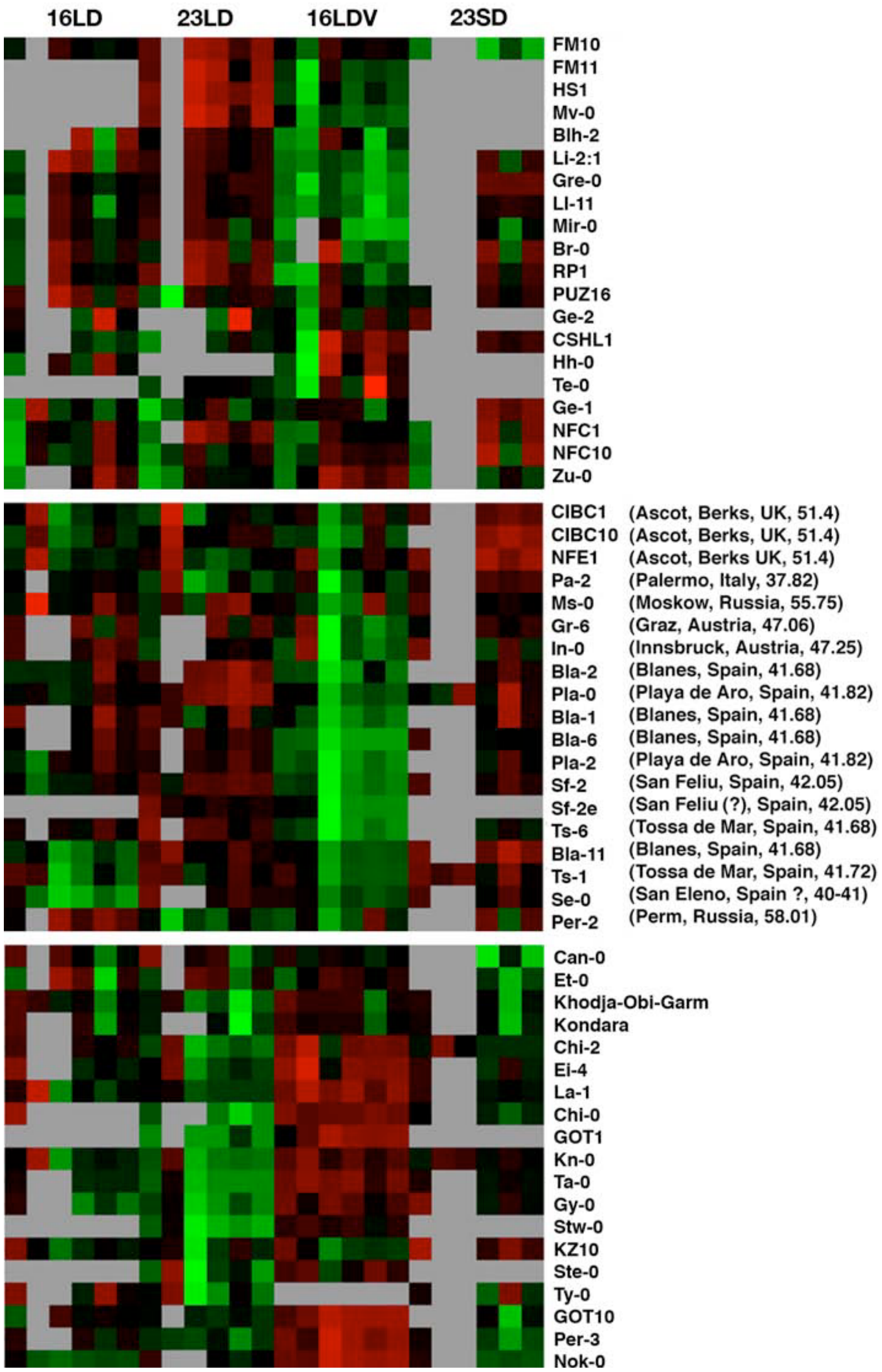

Supplementary Figure 5. Hierarchical clustering of accessions with functional *FRI/FLC* pathway.

Top, Accessions with moderate vernalization response. Middle, Accessions with extreme vernalization response. Several accessions in this cluster originate from lower latitudes (given in

parentheses). Bottom, Accessions that are delayed after vernalization, possibly indicating *FRI*-independent variation. Note that BG6, HR5 and Wil-1, which lack functional *FRI*, cluster with this group.
