# Supplementary material for: Diversity of Flowering Responses in Wild Arabidopsis thaliana Strains
Source: PLoS Genet. 2005 Jul 25;1(1):e6. doi: 10.1371/journal.pgen.0010006 (PMC1183525; doi:10.1371/journal.pgen.0010006)
Supplement: Table S1 — A, B, C, etc., denote multiple stocks and repeated assays. Latitude information was obtained from Geographic Names Information System (US Geological Survey). ALN, adult rosette leaf number; CLN, cauline leaf number; DTF, days to flowering; JLN, juvenile rosette leaf number; TLN, total leaf number. The data are also available as a CSV file (Dataset S1). (62 KB PDF) [file pgen.0010006.st001.pdf]

Supplementary Material

Supplementary Table 1. Means of flowering time traits in accessions and mutant strains (also available in CSV format).

A, B, C, etc. denote multiple stocks and repeated assays. Latitude information was obtained from Geographic Names Information System (US Geological Survey).

Traits: DTF, days to flowering; JLN, juvenile rosette leaf number; ALN, adult rosette leaf number; CLN, cauline leaf number; TLN, total leaf number.

Accessions							16ºC Long Day (16LD)						23ºC Long Day (23LD)						16ºC Long Day + Vernalization (16LDV)						23ºC Short Day (23SD)					
**No.**	**Ecotype**	**Stock No**	**Country**	**Latitude**	**Longitude**	***FRI*/*FLC* Status**	**DTF**	**JLN**	**ALN**	**RLN**	**CLN**	**TLN**	**DTF**	**JLN**	**ALN**	**RLN**	**CLN**	**TLN**	**DTF**	**JLN**	**ALN**	**RLN**	**CLN**	**TLN**	**DTF**	**JLN**	**ALN**	**RLN**	**CLN**	**TLN**
1	Ak-1	N939	Germany	48º04'	7º38' E	*fri*-Ler	43.45	8.80	23.00	32.57	7.43	40.00	21.64	3.18	12.45	15.64	4.08	20.00	24.82	7.00	12.55	19.55	4.82	24.36	44.58	6.36	29.18	35.55	7.91	43.45
2	Ang-1	N951	Belgium	50º37'	5º36' E	new *fri* allele	47.10	6.67	23.00	31.63	6.13	37.75	22.91	2.50	10.10	13.45	3.00	16.45	27.00	6.82	9.45	16.00	4.00	20.00	71.00	8.67	46.50	62.00	11.00	73.00
3	Bay-0	N955	Germany	49º56'53"	11º34'42" E	*fri*-Ler	25.75	NA	NA	13.13	3.13	16.25	16.17	3.58	5.17	8.75	2.33	11.08	21.82	7.00	5.40	12.45	2.73	15.18	49.29	8.33	27.33	36.27	6.64	42.91
4	Bch-3	N959	Germany	53º22'	10º34' E	*fri*-Col	33.18	11.80	7.30	19.36	6.55	25.91	21.75	5.75	9.83	15.58	5.33	20.92	27.40	12.00	6.82	18.82	5.91	24.73	56.78	9.57	32.14	41.45	10.45	51.91
5	Bd-0	N963	Germany	52º27'	13º17' E	new *flc* allele	28.73	8.45	7.09	15.55	4.55	20.09	18.25	4.00	8.36	12.36	4.18	16.55	21.00	9.00	4.60	13.60	3.40	17.00	48.10	8.60	31.50	40.18	10.82	51.00
6	BG1	N22341	USA	47º36'23"	122º19'51" W	new *fri* allele	43.00	8.00	12.00	19.75	6.25	26.00	19.58	4.14	9.29	13.58	4.00	17.58	22.18	6.73	10.55	17.27	5.82	23.09	58.30	NA	NA	60.33	13.33	73.67
7	BG4	N22344	USA	47º36'23"	122º19'51" W	new *fri* allele	38.80	NA	NA	21.00	7.00	28.00	20.30	3.00	8.80	17.67	4.42	22.08	22.00	6.00	8.64	14.64	4.36	19.00	67.33	NA	NA	58.67	13.89	72.56
8	BG6	N22346	USA	47º36'23"	122º19'51" W	new *fri* allele	39.44	8.50	15.00	35.20	9.70	44.90	23.78	2.86	13.71	20.00	6.11	26.11	24.25	6.75	12.50	19.25	6.75	26.00	62.75	NA	NA	70.56	16.00	86.56
9	BG9	N22349	USA	47º36'23"	122º19'51" W	new *fri* allele	38.43	NA	NA	18.71	5.71	24.43	21.50	2.89	9.56	12.60	3.20	15.80	22.60	6.00	7.44	13.70	4.30	18.00	63.91	NA	NA	62.11	16.00	78.56
10	Bla-1	N971	Spain	41º41'	2º48' E		56.00	NA	NA	42.00	12.86	54.29	44.22	5.75	28.83	37.63	10.67	43.33	21.92	6.17	5.00	11.17	3.50	14.67	NA	NA	NA	55.40	14.70	70.10
11	Bla-2	N973	Spain	41º41'	2º48' E		40.83	8.55	33.33	41.17	13.00	54.17	42.80	NA	46.73	52.17	12.64	60.64	18.67	6.17	4.42	10.58	4.25	14.83	NA	NA	NA	59.57	12.86	71.75
12	Bla-4	N977	Spain	41º41'	2º48' E	*fri*-Ler	28.50	6.75	4.63	12.08	4.00	16.08	18.45	4.50	5.40	9.90	3.60	13.50	22.50	7.33	5.25	12.58	4.67	17.25	46.11	9.36	27.73	37.09	10.00	48.00
13	Bla-5	N6620	Spain	41º41'	2º48' E		NA	NA	NA	NA	NA	NA	54.29	NA	45.60	43.13	9.20	54.80	16.20	6.56	1.11	7.67	3.44	11.11	NA	NA	NA	NA	NA	NA
14	Bla-6	N6621	Spain	41º41'	2º48' E		56.80	NA	NA	54.75	15.50	70.25	56.00	NA	52.71	47.63	12.00	64.71	21.00	6.83	5.17	12.00	3.33	15.33	91.00	NA	NA	69.50	12.75	82.25
15	Bla-11	N985	Spain	41º41'	2º48' E		28.17	6.92	13.58	20.50	5.83	26.33	34.75	6.30	25.92	28.08	7.91	37.18	16.42	5.27	3.27	8.55	2.27	10.82	67.20	NA	NA	58.64	14.82	73.45
16	Bla-12	N987	Spain	41º41'	2º48' E	*fri*-Col	27.70	8.38	6.50	14.20	3.10	17.30	18.83	4.27	6.91	11.18	2.45	13.64	22.58	9.25	5.58	14.83	3.17	18.00	61.88	NA	NA	55.91	10.73	66.64
17	Blh-2	N6657	Czech Republic	48º50'	16º45' E		NA	NA	NA	97.00	11.00	108.00	61.60	NA	69.50	69.50	13.25	82.75	27.89	8.78	17.00	25.78	4.67	30.44	NA	NA	NA	NA	NA	NA
18	Br-0	N995	Czech Republic	49º12'	16º38' E		38.67	NA	45.92	45.92	8.42	54.33	34.55	NA	46.17	46.33	7.08	53.25	18.00	NA	12.10	12.10	3.70	15.80	NA	NA	NA	69.44	8.33	77.78
19	Bs-1	N997	Switzerland	47º33'	7º35' E	*fri*-Ler	40.11	8.00	19.14	27.14	7.14	34.29	22.82	5.80	11.90	17.91	4.09	22.00	24.27	7.82	8.91	16.73	4.00	20.73	51.33	9.64	39.18	48.50	9.83	58.33
20	Bs-5	N1001	Switzerland	47º33'	7º35' E	*fri*-Col	42.10	8.63	22.88	29.30	6.80	36.10	20.33	2.33	11.67	14.00	3.83	17.83	23.75	6.33	11.25	17.58	4.50	22.08	46.00	6.55	28.82	35.75	7.08	42.83
21	Bsch-2	N1005	Germany	50º01'	8º40' E	*fri*-Ler	29.91	8.71	9.14	17.27	5.73	23.00	18.45	3.82	7.36	11.18	4.09	15.27	27.42	9.09	8.36	20.92	5.92	26.83	42.30	7.45	26.00	33.45	9.36	42.82
22	Bu-11	N1025	Germany	50º42'	9º43' E	*fri*-Col	31.33	9.67	6.89	16.56	3.78	20.33	17.70	5.50	6.50	12.00	3.20	15.20	23.08	10.08	5.67	15.75	3.67	19.42	60.40	9.50	40.25	57.09	13.09	70.55
23	Bur-0	N1029	Ireland	53º08'	9º04'32" W	new *flc* allele	47.86	NA	34.29	41.14	9.14	50.29	30.60	6.20	25.80	32.00	6.80	38.80	NA	NA	NA	NA	NA	NA	67.73	NA	NA	58.67	14.00	72.67
24	Cal-0	N1063	UK	53º10'	1º35' W		51.09	14.25	24.00	37.71	8.57	46.29	31.27	6.50	25.38	33.27	7.00	40.27	NA	16.63	17.50	34.13	7.63	41.75	57.09	NA	NA	51.43	9.86	61.29
25	Can-0	N1065	Spain	28º	15º30' W		58.73	NA	47.67	47.67	7.75	55.42	54.56	NA	41.13	47.00	6.38	47.50	26.00	7.60	10.00	17.60	4.40	22.00	NA	NA	NA	34.67	10.50	45.17
26	Cen-0	N1067	France	49º11	0º21' W	new *flc* allele	43.08	6.63	24.25	32.11	9.67	41.78	23.73	4.09	10.45	14.55	4.73	19.27	29.75	7.71	18.14	25.86	7.43	33.29	67.17	NA	NA	70.50	17.25	87.75
27	Cha-0	N1069	Switzerland	46º02'	7º07' E		73.13	NA	51.33	51.33	8.50	59.83	NA	NA	NA	NA	NA	NA	NA	NA	NA	NA	NA	NA	NA	NA	NA	73.40	6.40	79.80
28	Chi-0	N1073	Russia(?)				61.67	NA	NA	NA	NA	NA	34.33	NA	NA	32.44	5.56	38.00	28.82	8.91	10.82	19.73	5.64	25.36	63.82	NA	NA	56.88	9.13	66.00
29	Chi-2	N1077	Russia(?)				52.57	NA	NA	44.75	8.50	53.25	26.09	7.27	13.91	21.18	5.36	26.55	32.11	15.56	6.44	22.00	6.44	28.44	51.92	14.60	29.80	43.83	7.83	51.67
30	CIBC1	N22220	UK	51º24'	0º40' W		43.55	13.30	24.40	37.70	9.10	46.80	31.20	8.90	24.80	36.45	8.20	41.90	21.17	7.75	5.63	13.38	5.00	18.38	69.83	NA	NA	66.17	12.83	79.00
31	CIBC10	N22229	UK	51º24'	0º40' W		43.14	12.00	31.17	41.17	10.67	51.83	40.18	8.13	38.73	41.75	9.45	54.09	21.36	7.29	6.43	13.71	5.14	18.86	NA	NA	NA	79.20	15.50	94.70
32	Co-1	N1085	Portugal	40º12'	8º25' W		31.70	NA	NA	18.73	5.00	23.73	26.64	NA	NA	22.00	7.30	29.30	17.55	9.00	0.50	9.56	2.20	11.70	58.50	NA	NA	35.00	9.82	44.82
33	CSHL1	N22419	USA	43º40'15"	74º20'37" W		58.09	NA	NA	52.67	10.33	63.00	37.08	NA	NA	45.75	11.25	57.00	28.83	5.50	17.80	23.45	7.45	30.91	NA	NA	NA	84.60	14.20	98.80
34	Cvi-0	N1097	Cape Verde	16º	24º W	*FRI* non-functional	34.80	5.60	7.80	13.40	2.80	16.20	39.00	4.20	9.00	13.56	3.22	16.78	21.09	4.64	3.55	8.18	1.82	10.00	55.25	5.86	9.86	17.75	3.63	21.38
35	Db-0	N1101	Germany			*fri*-Col	31.50	6.50	11.83	18.33	3.83	22.17	22.80	3.11	8.67	11.78	2.67	14.44	26.75	7.25	10.92	18.17	3.92	22.08	53.80	6.17	30.17	36.36	8.09	44.45
36	Di-1	N1109	France	47º25	5º E	*fri*-Ler	28.17	9.58	5.33	14.92	3.33	18.25	18.45	5.58	5.17	10.75	2.58	13.33	21.36	8.55	3.64	12.18	2.36	14.55	48.20	10.45	27.36	37.82	7.64	45.45
37	Dr-0	N1115	Germany	51º03'	13º44' E	*fri*-Ler	30.67	10.75	2.75	13.50	3.08	16.58	18.00	4.83	5.33	10.17	2.17	12.33	25.82	11.45	4.27	15.73	3.55	19.27	52.88	12.10	28.20	40.42	8.75	49.17
38	Dra-1	N1119	Czech Republic	49º25'	16º16' E	*fri*-Ler	31.29	12.11	10.44	22.56	4.33	26.89	19.17	5.92	7.42	13.08	3.08	16.17	24.73	9.08	9.67	18.75	4.00	22.75	36.33	10.58	18.75	29.33	5.33	34.67
39	Ei-4	N1127	Germany	50º15'	6º40' E		56.75	NA	NA	29.20	7.20	36.40	29.50	6.14	16.00	23.64	5.64	29.27	29.63	13.78	6.44	20.22	5.33	25.56	56.00	NA	NA	37.14	9.29	46.43
40	Ei-6	N1131	Germany	50º15'	6º40' E	*fri*-Col	22.18	5.63	6.63	11.30	3.50	14.80	16.36	2.42	6.25	8.67	2.58	11.25	19.60	5.55	6.82	12.36	3.64	16.00	38.20	5.60	16.10	21.70	5.30	27.00
41	El-0	N1135	Germany	51º18'	9º58' E	*fri*-Col	25.58	7.30	4.60	11.75	2.92	14.67	16.25	3.91	6.27	10.18	3.27	13.45	22.42	8.17	6.17	14.33	3.25	17.58	51.71	11.00	27.25	35.78	8.67	44.44
42	En-1	N1137	Germany	50º09'	8º46' E	*fri*-Ler	30.20	8.50	7.50	16.50	4.00	20.50	17.33	3.36	7.00	10.36	3.55	13.91	20.80	6.50	6.00	12.50	4.00	16.50	35.58	6.67	10.11	18.91	5.27	24.18
43	Enk-T	N6176	Tajikistan			*fri*-Col	28.20	8.67	5.67	14.10	5.60	19.70	19.30	5.70	6.50	12.20	3.90	16.10	25.58	9.09	5.00	14.09	4.91	19.00	62.88	NA	NA	43.55	13.55	57.09
44	Er-0	N1143	Germany	49º35'23"	11º E	*fri*-Ler	45.30	8.71	38.29	47.00	8.63	55.63	23.64	4.55	19.55	24.09	5.55	29.64	24.33	8.83	10.92	19.75	4.36	23.75	77.40	NA	NA	88.57	12.86	101.43
45	Est-1	N1151	Estonia	59º	26º E	*fri*-Col	28.58	6.67	6.67	13.67	4.17	17.83	17.91	4.00	6.36	10.36	3.73	14.09	28.17	9.45	10.64	20.08	6.25	26.33	53.50	NA	NA	28.20	9.50	37.70
46	Et-0	N1153	France	44º38'	3º E		55.71	NA	74.63	74.63	8.75	83.38	49.75	NA	63.70	64.60	9.80	74.30	26.90	11.00	14.90	25.90	6.40	32.30	NA	NA	NA	78.00	10.25	88.25
47	Fe-1	N1155	Germany	48º00'29"	7º49'57" E	*fri*-Ler	40.00	NA	NA	20.00	6.20	26.20	26.00	4.67	10.56	15.22	4.33	19.56	28.56	8.33	7.56	15.89	5.78	21.67	48.78	9.09	20.09	28.75	10.75	39.50
48	Fi-0	N1157	Germany	50º30'	8º01' E	*fri*-Ler	60.50	NA	NA	NA	NA	NA	37.17	NA	NA	36.44	6.44	42.89	32.00	8.88	11.13	20.00	3.50	23.50	96.17	NA	NA	62.75	9.50	72.25
49	FM10	N22391	USA	42º26'26"	76º29'49" E		48.90	NA	50.22	50.22	10.22	60.44	55.86	NA	67.25	67.25	11.00	78.25	25.08	6.45	12.00	18.45	5.36	23.82	50.91	NA	NA	36.73	9.91	46.64
50	FM11	N22392	USA	42º26'26"	76º29'49" E		NA	NA	NA	NA	NA	NA	52.11	NA	62.00	62.00	9.88	71.88	24.91	5.91	11.27	17.18	4.91	22.09	NA	NA	NA	NA	NA	NA
51	Fr-2	N1169	Germany	50º07'	8º41' E	*fri*-Col	26.91	6.45	7.73	14.18	3.36	17.55	18.83	2.92	8.42	11.33	3.42	14.75	23.00	6.42	8.25	14.67	4.33	19.00	33.22	6.00	14.27	20.27	6.09	26.36
52	Fr-4	N1173	Germany	50º07'	8º41' E	*fri*-Ler	32.80	7.67	7.44	15.11	4.22	19.33	19.50	3.90	6.60	10.45	3.82	14.27	27.00	8.50	6.33	14.83	4.83	19.67	57.22	8.20	39.80	47.55	9.91	57.45
53	Fr-6	N1177	Germany	50º07'	8º41' E	*fri*-Col	26.75	NA	NA	12.42	3.33	15.75	17.67	5.75	5.50	11.25	3.50	14.75	21.83	9.83	4.50	14.33	3.25	17.58	52.91	11.43	45.14	58.40	12.20	70.60
54	Ga-0	N1181	Germnay	50º25	8º03' E	*fri*-Ler	42.44	12.14	23.00	35.13	6.50	41.63	23.30	6.10	14.90	21.64	4.00	25.64	24.27	9.45	9.27	18.73	3.82	22.55	64.40	11.00	45.00	60.33	8.89	69.22
55	Gd-1	N1185	Germany	53º33'	10º47' E	*fri*-Col	27.78	5.88	3.75	9.78	2.44	12.22	18.00	4.00	5.91	9.91	3.00	12.91	21.33	6.75	3.83	10.58	2.67	13.25	44.64	7.11	21.67	29.10	7.20	36.30
56	Ge-1	N1189	Switzerland	46º12'	6º10' E		44.92	13.90	41.50	53.08	10.08	63.17	34.83	5.70	42.67	51.08	8.75	56.17	25.17	9.09	10.82	19.91	4.55	24.45	NA	NA	NA	79.75	13.50	93.25
57	Ge-2	N1191	Switzerland	46º12	6º10' E		68.78	NA	NA	55.00	20.00	75.00	NA	NA	NA	47.20	18.60	65.80	32.00	7.63	13.63	21.25	7.13	28.38	102.00	NA	NA	NA	NA	NA
58	Gö-2	N1197	Germany	51º30'	9º54' E	*fri*-Col	29.44	6.43	13.29	19.11	6.22	25.33	22.42	3.67	15.17	18.83	6.17	25.00	25.58	6.92	11.00	17.92	6.33	24.25	42.64	7.80	28.00	37.00	9.55	46.55
59	GOT1	N22277	Germnay	51º30'	9º54' E		NA	NA	NA	NA	NA	NA	44.27	NA	39.90	43.33	11.20	53.50	33.00	12.92	18.00	30.92	8.33	39.25	NA	NA	NA	NA	NA	NA
60	GOT10	N22286	Germnay	51º30'	9º54' E		50.88	NA	57.63	57.63	13.25	70.88	50.08	NA	45.64	46.82	10.73	57.09	34.83	12.83	18.67	31.50	8.58	40.08	NA	NA	NA	79.00	8.40	87.40
61	Gr-1	N1199	Austria	47º04	15º24' E	*fri*-Ler	49.00	NA	NA	NA	NA	NA	22.92	4.83	13.00	17.83	3.33	21.58	26.50	10.73	10.09	20.82	4.27	25.09	47.90	9.73	27.45	37.18	6.45	43.64
62	Gr-3	N1203	Austria	47º04	15º24' E	*flc*-Da1	42.14	9.50	17.67	26.50	5.13	31.63	21.67	4.08	10.50	14.58	3.50	18.08	28.00	7.73	12.09	19.82	5.00	24.82	45.00	7.25	32.75	37.58	7.83	45.42
63	Gr-6	N6728	Austria	47º04	15º24' E		59.82	NA	NA	62.33	9.50	71.83	40.10	NA	NA	55.33	8.75	64.08	24.09	11.00	5.00	16.00	3.64	19.64	69.10	NA	NA	73.70	12.10	85.80
64	Gre-0	N1211	USA	43º10'39"	85º15'10" W		59.00	NA	62.00	62.00	12.25	74.25	56.63	NA	61.43	56.71	13.00	74.43	24.44	5.86	10.86	16.71	3.86	20.57	NA	NA	NA	102.50	18.25	120.75
65	Gü-0	N1213	Germany	50º24'	8º01' E	*fri*-Col	30.67	8.20	6.40	14.50	4.70	19.20	18.60	4.64	8.18	12.82	4.36	17.18	25.75	9.92	6.50	16.42	4.83	21.25	42.50	NA	NA	30.27	10.09	40.36
66	Gy-0	N1217	France	48º46'	2º5' E		38.55	NA	NA	20.00	6.00	26.00	23.00	4.11	11.67	16.45	4.73	21.18	24.25	5.90	9.80	15.67	3.42	19.08	70.18	NA	NA	39.00	8.83	47.83
67	Hau-0	N1221	Denmark	55º40'	12º35' E	*fri*-Ler	25.08	6.70	5.80	12.25	3.67	15.92	17.33	3.67	6.83	10.50	3.92	14.42	20.27	6.80	6.30	13.10	3.90	17.00	45.50	6.89	30.11	36.82	9.36	46.18
68	Hh-0	N1225	Germany	54º25	9º53' E		56.45	NA	59.08	59.08	17.58	76.67	NA	NA	NA	NA	NA	NA	29.44	6.78	17.70	23.80	8.30	32.10	NA	NA	NA	NA	NA	NA
69	Hl-0	N1229	Germany	52º23'	9º29' E	*fri*-Col	27.83	8.67	6.00	13.75	3.42	17.17	22.09	5.64	11.55	17.18	3.82	21.00	24.36	9.82	7.55	17.36	4.00	21.36	51.00	9.50	27.88	37.60	7.40	45.00
70	Hl-3	N1233	Germany	52º23'	9º29' E	*fri*-Ler	24.33	5.50	4.80	10.09	3.18	13.27	17.17	4.08	6.25	10.33	3.42	13.75	20.58	6.42	5.33	11.75	3.00	14.75	39.09	8.42	20.17	28.58	9.33	37.92
71	Khodja-Obi	N6178	Tajikistan	39º	68º30' E		51.82	9.60	27.40	38.38	4.63	43.00	28.91	5.13	17.88	25.27	2.36	27.64	27.00	7.45	8.73	16.18	2.27	18.45	58.33	NA	NA	48.91	3.82	52.73
72	HR15	N22214	UK	51º24'	0º40' W	new *fri* allele	35.50	7.71	14.86	22.00	6.13	28.13	26.18	4.22	18.22	22.09	7.55	29.64	22.90	7.70	6.90	14.60	5.20	19.80	53.27	9.80	41.90	51.92	13.25	65.17
73	HR5	N22205	UK	51º24'	0º40' W	new *fri* allele	28.55	8.50	9.80	17.82	4.91	22.73	23.09	4.09	15.18	19.27	5.36	24.64	20.73	7.17	8.42	15.58	5.00	20.58	49.00	7.80	41.90	50.36	12.00	62.36
74	Hs-0	N1237	Germany	52º32'	8º42' E		28.80	7.86	6.57	14.20	5.30	19.50	16.58	4.67	6.17	10.83	3.92	14.75	21.92	8.00	5.83	13.83	4.42	18.25	41.63	10.10	25.20	35.36	9.00	44.36
75	HS1	N22351	USA	42º22'25"	71º07'12" W		NA	NA	NA	NA	NA	NA	60.14	NA	68.43	65.71	12.57	81.00	24.42	5.67	11.00	16.67	5.22	21.89	NA	NA	NA	NA	NA	NA
76	In-0	N1239	Austria	47º15'	11º24' E		58.70	NA	NA	54.43	12.86	67.29	41.50	NA	NA	46.40	10.10	56.50	24.00	10.64	6.91	17.55	4.45	22.00	85.13	NA	NA	63.43	13.57	77.00
77	Is-0	N1241	Germany	50º29'	7º36' E	*fri*-Ler	25.00	9.64	2.55	12.18	3.36	15.55	17.50	5.75	4.83	10.58	3.33	13.92	21.25	10.42	3.25	13.67	3.33	17.00	46.25	13.30	21.60	35.45	10.27	45.73

78	Jl-1	N1249	Czech Republic	50º06'	12º41' E	new *fri* and *flc* alleles	40.33	10.22	16.67	26.89	7.11	34.00	18.75	4.33	7.33	11.67	3.75	15.42	23.91	9.64	6.91	16.55	3.82	20.36	48.33	9.20	29.90	38.82	9.73	48.55
79	Jm-1	N1261	Czech Republic	49º04'	16º15' E	*fri*-Ler	31.27	11.50	8.50	18.70	5.60	24.30	17.50	4.42	5.25	9.67	3.67	13.33	20.92	8.75	5.08	13.83	4.25	18.08	37.17	10.00	15.20	25.20	8.20	33.40
80	Kil-0	N1271	UK	55º38'	5º39' W	*fri*-Col	26.89	10.25	4.00	14.00	4.78	18.78	20.09	7.45	5.73	13.18	4.64	17.82	22.75	10.11	5.11	15.22	5.00	20.22	45.38	13.75	21.25	35.00	10.88	45.88
81	Kin-0	N1273	USA	43º22'02	85º18'05" W	*FRI* non-functional	38.11	6.67	10.17	17.11	4.00	21.11	20.67	2.78	11.00	13.90	4.00	17.90	29.22	6.82	9.45	16.27	4.09	20.36	49.38	NA	NA	30.25	6.13	36.38
82	Kl-0	N1275	Germany	50º56'	6º57' E	*fri*-Col	26.44	9.63	3.50	12.89	4.22	17.11	19.36	7.36	6.27	13.64	4.09	17.73	22.09	11.18	4.36	15.55	4.91	20.45	42.90	13.64	22.64	36.27	10.36	46.64
83	Kn-0	N1287	Lithuania	54º54'	23º54' E		37.30	14.75	15.33	30.08	7.08	37.17	21.92	5.83	12.92	18.75	4.17	22.92	24.27	10.25	8.08	18.33	4.00	22.33	44.30	10.45	32.64	43.09	9.36	52.45
84	Kondara	N6175	Tajikistan	38º48'43"	68º49' E		59.60	NA	NA	50.00	5.00	55.00	34.36	NA	NA	36.55	2.64	39.18	27.11	7.90	9.60	17.50	3.10	20.60	65.50	NA	NA	55.86	4.71	60.57
85	KZ10	N22442	Kazakhstan	50º25'	55º36' E		38.75	6.00	18.00	25.57	6.14	31.71	24.40	3.57	12.43	22.10	5.70	27.80	17.91	4.82	4.09	8.91	2.55	11.45	58.45	NA	NA	40.00	8.73	48.73
86	La-1	N1303	Poland	52º44'	15º14' E		47.91	19.56	20.11	40.10	6.90	47.00	36.18	5.29	24.57	29.30	6.20	35.50	30.70	14.44	12.78	27.22	5.56	32.78	68.00	NA	NA	49.27	9.36	58.64
87	Li-2:1	N1315	Germany	50º23'	8º03' E		50.08	NA	70.50	71.75	9.08	80.83	53.80	NA	54.89	54.88	10.44	66.22	22.33	7.17	10.00	17.17	3.67	20.83	NA	NA	NA	85.00	11.17	96.17
88	Lip-0	N1337	Poland	53º28'	21º08' E	*FLC* non-functional	28.25	7.30	7.20	14.67	3.83	18.50	17.25	4.18	7.18	11.36	3.00	14.36	21.67	6.92	6.33	13.25	3.42	16.67	41.33	8.22	23.44	31.20	11.10	42.30
89	Ll-11	N1341	Spain	41º49'	2º54' E		49.17	NA	66.58	67.00	9.08	76.08	60.40	NA	63.75	56.22	12.25	76.00	23.58	7.00	10.88	17.88	3.38	21.25	NA	NA	NA	88.00	16.60	104.60
90	Ll-2	N1343	Spain	41º49'	2º54' E	new *flc* allele	23.00	5.50	1.00	6.40	2.80	9.20	15.82	4.20	2.60	6.82	3.09	9.91	15.73	4.64	1.82	6.45	2.64	9.09	29.75	5.42	7.00	12.42	4.83	17.25
91	Lö-1	N1347	Germany	47º37'	7º40' E	*fri*-Ler	38.50	6.36	22.27	28.64	9.55	38.18	21.82	2.91	13.55	16.45	4.91	21.36	26.64	7.45	15.55	23.00	7.55	30.55	46.50	6.92	37.92	44.83	11.25	56.08
92	M7323S	N6184				*fri*-Col	39.89	9.80	15.60	27.10	5.80	32.90	24.40	3.75	9.88	13.89	2.67	16.56	24.17	7.50	6.60	14.42	4.00	18.42	80.63	NA	NA	53.33	6.67	60.00
93	M7884S	N6188	USA	47º36'23"	122º19'51" E	new *fri* allele	36.30	5.89	19.22	25.11	6.00	31.11	25.00	2.00	13.33	15.30	3.90	19.20	21.58	4.50	8.75	13.25	3.33	16.58	61.00	5.56	51.33	59.08	10.50	69.58
94	Mc-0	N1363	UK	54º36'	2º18' W	*fri*-Col	48.50	13.43	17.71	31.14	9.57	40.71	22.25	5.33	11.25	16.58	6.17	22.75	29.64	10.67	12.83	23.50	8.17	31.67	52.00	8.82	44.82	53.64	15.27	68.91
95	Mh-1	N1369	Poland	53º47'	20º29' E	*fri*-Col	29.91	8.63	7.75	16.55	3.09	19.64	23.78	3.78	6.89	10.67	2.89	13.56	22.58	7.08	7.00	14.08	3.25	17.33	53.30	9.22	37.78	47.00	13.80	60.80
96	Mir-0	N1379	Italy	45º38'55"	13º46'48" E		54.42	NA	63.25	63.25	11.67	74.92	62.22	NA	61.56	61.38	9.00	70.56	20.00	NA	13.20	13.20	3.40	16.60	NA	NA	NA	81.30	9.70	91.00
97	Ms-0	N1377	Russia	55º45'08"	37º36'56" E		41.73	14.80	34.18	40.91	11.09	52.00	33.33	5.56	30.17	44.58	9.92	44.25	21.64	6.50	5.88	12.38	5.25	17.63	66.71	NA	NA	54.00	10.13	64.13
98	Mv-0	N1387	USA	41º27'17"	70º33'42" W		NA	NA	NA	NA	NA	NA	47.45	NA	56.30	55.90	9.11	66.11	22.92	5.57	8.29	13.86	4.00	17.86	NA	NA	NA	NA	NA	NA
99	Mz-0	N1383	Germany	50º51	8º29' E	*fri*-Col	29.22	NA	NA	11.56	3.00	14.56	17.91	4.73	5.91	10.64	2.73	13.36	23.78	7.50	6.38	13.78	3.44	17.22	79.00	NA	NA	46.29	11.14	57.43
100	Nc-1	N1389	France	48º37'	6º15' E	*fri*-Ler	31.00	9.91	9.82	19.00	6.17	25.17	17.73	5.27	7.55	12.82	4.00	16.82	24.00	9.64	6.45	16.09	5.18	21.27	62.30	NA	NA	62.80	14.80	77.60
101	Nd-1	N1680	Germany	50º28'	8º02' E	*fri*-Col	27.64	5.80	6.10	11.91	2.45	14.36	18.18	4.42	6.42	10.83	2.67	13.50	22.75	7.42	5.50	12.92	3.17	16.08	44.20	8.56	24.44	33.00	11.11	44.11
102	NFC1	N22182	UK	51º24'	0º40' W		44.70	13.20	48.90	55.50	15.10	70.60	42.75	NA	62.67	62.18	11.42	74.83	25.18	8.33	12.89	21.22	5.89	27.11	68.33	NA	NA	95.80	12.80	108.40
103	NFC10	N22191	UK	51º24'	0º40' W		44.11	13.57	45.33	55.89	15.33	71.22	40.17	6.60	53.82	48.50	11.00	67.82	24.90	9.40	13.50	22.90	6.20	29.10	71.00	NA	NA	102.73	12.00	114.73
104	NFE1	N22163	UK	51º24'	0º40' W		41.00	12.67	31.00	40.50	8.42	48.92	37.92	6.70	32.17	31.18	7.00	44.75	21.45	8.56	6.44	15.00	4.56	19.56	71.50	NA	NA	74.73	12.00	86.73
105	Nok-0	N1399	Netherlands	52º14'	4º26' E		49.27	7.60	27.40	35.33	8.00	43.33	46.00	NA	NA	41.00	7.50	48.86	31.67	10.11	18.44	28.56	7.44	36.00	70.86	NA	NA	46.00	8.58	54.58
106	Nok-1	N1401	Netherlands	52º14'	4º26' E		43.00	7.86	31.50	37.00	8.20	45.20	41.83	3.60	34.44	39.56	7.78	44.22	27.91	7.64	15.09	22.73	6.09	28.82	NA	NA	NA	46.63	8.50	55.13
107	Nok-3	N1405	Netherlands	52º14'	4º26' E		41.27	7.40	31.25	37.42	8.83	46.25	42.18	NA	37.64	36.55	8.18	46.55	26.64	8.00	12.64	20.64	6.27	26.91	70.88	NA	NA	44.83	8.58	53.42
108	Np-0	N1397	Germany	52º41'	10º57' E	*fri*-Col	30.10	7.14	7.86	14.40	3.80	18.20	17.25	4.17	5.00	9.17	3.08	12.25	22.42	7.58	5.25	12.83	3.17	16.00	39.50	8.44	12.00	20.90	5.40	26.30
109	Nw-1	N1411	Germany	50º19'	8º25' E	*fri*-Ler	27.00	NA	NA	13.89	5.22	19.11	17.91	3.50	10.20	13.64	5.82	19.45	20.50	6.22	7.44	13.40	5.20	18.60	44.82	NA	NA	37.78	11.00	48.78
110	Nw-3	N1415	Germany	50º15'	8º15' E	*fri*-Col	26.00	6.75	5.50	12.82	3.73	16.55	16.83	3.75	7.42	11.17	4.17	15.33	17.71	5.83	4.67	10.50	3.83	14.33	41.25	NA	NA	29.22	9.33	38.56
111	Ob-1	N1421	Germany	50º12'	8º35' E	*fri*-Col	30.44	5.11	6.44	11.40	4.00	15.40	19.09	2.36	6.27	8.64	3.64	12.27	23.83	7.00	5.58	12.58	3.83	16.42	51.90	5.44	31.22	37.10	10.00	47.10
112	Ob-3	N1425	Germany	50º12'	8º35' E	*fri*-Col	26.43	8.29	4.71	13.00	3.14	16.14	19.50	5.25	6.42	11.67	3.17	14.83	22.64	8.27	5.82	14.09	3.82	17.91	50.80	9.00	30.33	39.33	11.50	50.83
113	Old-2	N1429	Germany	53º02'14"	8º21' E	*fri*-Col	29.33	8.67	5.67	14.00	4.67	18.67	22.33	5.25	9.08	14.33	4.75	19.08	24.42	9.58	6.50	16.08	5.00	21.08	44.56	10.00	22.22	32.55	9.64	42.18
114	Ove-0	N1435	Germany	53º20'	8º25' E	*fri*-Col	32.33	7.25	12.75	21.33	5.17	26.50	22.75	2.71	14.00	17.33	4.44	21.78	27.70	7.00	12.50	19.50	5.30	24.80	48.00	NA	NA	26.33	7.43	33.67
115	Pa-2	N1441	Italy	37º49'	13º35' E		44.17	NA	33.20	41.00	10.00	51.00	28.42	9.08	15.67	23.08	8.42	33.17	19.10	8.60	2.30	10.90	4.40	15.30	68.33	NA	NA	75.17	12.25	87.58
116	Per-2	N1449	Russia	58º01'	56º10' E		50.83	NA	52.33	52.33	12.75	65.08	40.67	4.00	36.25	36.42	9.08	47.00	21.33	8.33	7.11	15.44	5.67	21.11	NA	NA	NA	73.70	9.80	83.50
117	Per-3	N1451	Russia	58º01'	56º10' E		48.88	NA	NA	46.60	8.20	54.80	33.40	4.40	29.20	36.10	5.40	40.50	29.27	8.36	16.18	24.55	5.91	30.45	62.88	NA	NA	48.50	6.38	54.88
118	Pi-0	N1455		47º07'	10º47' E	*fri*-Ler	37.63	9.14	13.86	23.63	5.13	28.75	22.91	3.64	11.45	15.09	3.27	18.36	27.11	8.00	10.25	18.22	4.11	22.33	55.44	NA	NA	34.10	7.90	41.80
119	Pla-0	N1459	Spain	41º49'	3º04' E		44.57	10.00	33.00	41.50	11.13	52.63	38.00	6.11	49.44	55.73	12.82	68.55	22.18	7.64	4.82	12.45	3.73	16.18	62.25	8.67	48.67	55.75	17.00	72.75
120	Pla-2	N1463	Spain	41º49'	3º04' E		44.83	6.29	44.92	48.58	13.83	62.42	46.60	NA	45.33	45.83	12.00	57.83	17.25	6.33	3.42	9.75	3.25	13.00	NA	NA	NA	57.17	15.17	72.33
121	Pog-0	N1477	Canada	49º40'	125º50' W	new *fri* allele	44.20	9.40	21.20	30.33	6.50	36.83	22.33	5.00	11.27	16.25	3.17	19.42	22.91	9.09	7.82	16.91	3.36	20.27	78.55	NA	NA	78.50	8.50	88.25
122	PUZ16	N22451	Czech Republic	49º25'	16º21' E		56.17	NA	54.75	55.92	10.17	66.08	39.33	4.00	44.83	42.92	9.67	56.83	24.27	6.86	11.29	18.14	4.71	22.86	67.50	NA	NA	70.18	11.64	81.82
123	Ra-0	N1481	France	46º01'	3º22' E	*fri*-Ler	28.22	8.83	3.50	12.11	3.78	15.89	17.75	4.25	2.75	7.00	3.50	10.50	21.60	8.78	4.00	12.78	3.89	16.67	53.25	10.33	31.00	41.80	9.30	51.10
124	REN1	N22253	France	47º01'	5º51' E	*fri*-Ler	41.88	8.33	21.17	28.50	6.63	35.13	28.11	4.88	13.25	18.13	4.25	22.38	24.18	7.36	6.82	14.18	3.73	17.91	57.64	8.29	42.14	54.25	9.50	63.75
125	Ri-0	N1493	Canada	49º10'	122º58' W	new *fri* allele	37.00	4.38	18.25	22.63	6.38	29.00	28.00	2.56	14.67	17.22	4.33	21.22	21.70	5.73	6.73	12.45	3.27	15.73	70.30	3.75	55.63	61.50	10.92	72.42
126	RLD1	N913				*fri*-Col	25.11	NA	NA	9.00	2.63	11.63	15.50	3.63	3.88	7.50	2.13	9.63	19.83	6.17	3.83	10.00	2.75	12.75	28.91	4.91	7.27	12.18	3.27	16.36
127	RP1	N22362	USA	42º26'26"	76º29'49" E		63.57	NA	66.86	66.86	14.29	81.14	66.67	NA	68.67	68.67	13.17	81.83	26.67	8.75	14.75	23.50	5.75	29.25	NA	NA	NA	96.75	15.25	112.00
128	Rsch-0	N1491	Russia	56º6'	35.91 E	*FLC* non-functional	31.42	7.45	14.09	22.25	4.00	26.25	19.92	3.83	10.92	14.75	2.83	17.58	20.27	5.36	6.64	12.00	3.09	15.09	48.67	7.44	43.00	50.44	6.33	56.78
129	Se-0	N1503	Spain				42.83	5.50	14.00	19.50	5.75	25.25	48.57	NA	NA	36.00	8.80	44.80	20.80	6.67	4.67	11.33	2.83	14.17	67.71	NA	NA	48.13	11.63	59.75
130	Sf-2	N1517	Spain	42º03'	2º16' E		37.67	5.08	27.33	32.42	8.75	41.17	39.67	3.86	38.75	40.44	9.17	50.17	14.25	5.08	2.42	7.50	2.33	9.83	63.83	NA	NA	49.17	11.08	61.00
131	Sf-2e	N1675					NA	NA	NA	NA	NA	NA	30.33	3.55	22.83	26.27	5.25	31.33	14.00	4.58	2.83	7.42	2.08	9.50	NA	NA	NA	NA	NA	NA
132	Sg-1	N1519	Germany	48º7'	8º20' E	*fri*-Ler	34.10	5.80	14.60	20.40	5.70	26.10	20.80	2.73	11.18	13.91	4.64	18.55	23.70	5.80	8.80	14.60	4.40	19.00	49.22	6.50	32.90	39.40	9.10	48.50
133	Shahdara	N6180	Tajikistan	37º29'	71º33' E	*FLC* non-functional	34.18	6.50	9.30	15.80	3.30	19.10	18.20	4.00	4.56	8.50	2.30	10.80	20.82	5.27	3.55	8.82	2.73	11.55	41.00	6.44	13.33	21.91	4.18	26.09
134	Sn(5)-1	N6181	Czech/Slovakia			*fri*-Ler	29.82	9.90	7.90	17.73	3.27	21.00	19.10	4.82	8.18	23.91	3.00	26.91	28.67	9.67	9.17	18.83	4.08	22.92	45.75	10.25	23.38	33.63	7.50	41.13
135	SQ4	N22243	UK	51º24'	0º40' W		30.78	6.00	7.80	15.78	4.44	20.11	19.75	4.60	7.40	12.00	2.80	14.80	23.83	8.00	6.08	14.08	3.75	17.83	56.00	8.75	43.00	52.20	9.50	61.70
136	Ste-0	N1537	Germany	52º36'	11º51' E		NA	NA	NA	NA	NA	NA	28.33	7.00	15.57	31.57	7.29	29.86	23.71	8.50	7.00	15.50	5.25	20.75	NA	NA	NA	NA	NA	NA
137	Stw-0	N1539	Russia	52º57'	36.08 E	new *flc* allele	NA	NA	NA	NA	NA	NA	22.29	4.71	10.14	14.86	3.71	18.57	21.67	8.00	8.25	16.25	3.50	19.75	NA	NA	NA	NA	NA	NA
138	Su-0	N1541	UK	53º40'	3º W		NA	NA	NA	NA	NA	NA	46.40	8.50	44.90	48.22	10.20	58.50	26.00	10.92	10.67	21.58	6.33	27.92	NA	NA	NA	NA	NA	NA
139	Ta-0	N1549	Czech Republic	49º25'	14º40' E		43.50	NA	NA	34.83	7.67	42.50	25.75	5.00	17.17	22.17	4.67	26.83	27.33	10.33	11.17	21.50	4.50	26.00	59.83	NA	NA	48.17	9.67	57.83
140	Te-0	N1551	Finland	60º04	23º18' E		NA	NA	NA	NA	NA	NA	44.33	NA	45.00	48.25	10.27	56.91	25.36	7.00	11.73	18.73	7.64	26.36	NA	NA	NA	NA	NA	NA
141	Ts-1	N1553	Spain	41º43'	2º56' E		44.80	8.75	15.00	23.80	7.20	31.00	39.14	5.25	24.13	29.89	7.89	37.78	20.91	5.73	4.00	9.73	2.64	12.36	66.00	9.00	34.50	43.00	11.67	54.67
142	Ts-6	N1561	Spain	41º43'	2º56' E		45.78	NA	37.44	38.22	11.44	49.67	57.67	NA	44.75	46.75	7.50	52.25	17.55	5.36	2.64	8.00	2.64	10.64	NA	NA	NA	45.70	10.30	56.00
143	Tsu-1	N6926	Japan	34º43'	136º31' E	*fri*-Ler	39.43	12.67	19.00	31.67	8.67	40.33	21.55	5.64	11.09	16.73	4.82	21.55	30.70	12.64	18.45	31.09	8.55	39.64	39.89	10.09	25.55	35.64	10.91	46.55
144	Ty-0	N1573	UK	56º25'	5º14' W		56.20	NA	NA	35.22	12.33	47.56	32.40	6.50	14.33	22.20	7.20	29.40	NA	NA	NA	NA	NA	NA	56.60	NA	NA	38.40	12.70	52.10
145	Uk-1	N1575	Germany	48º02'	7º46' E	*fri*-Ler	54.00	9.25	51.50	62.00	13.40	75.40	36.45	5.86	33.14	45.36	11.09	56.45	30.80	10.67	16.00	26.67	7.78	34.44	NA	NA	NA	79.33	14.83	94.17
146	Uk-2	N1579	Germany	48º02'	7º46' E	*fri*-Ler	29.42	6.29	4.71	10.67	3.92	14.58	23.75	4.10	6.90	11.00	3.90	14.90	22.89	6.88	5.25	12.13	3.38	15.50	44.00	6.63	17.63	24.00	9.44	33.44
147	Uk-3	N1577	Germany	48º02'	7º46' E	*fri*-Ler	40.90	10.88	16.63	27.50	4.38	31.88	27.30	6.56	21.00	26.30	4.40	30.70	24.60	9.27	8.09	17.36	3.45	20.82	55.70	11.25	32.50	43.75	7.13	50.88
148	Uk-4	N1581	Germany	48º02'	7º46' E	*fri*-Ler	31.91	9.89	9.78	19.30	5.00	24.30	20.27	5.00	10.73	15.73	4.36	20.09	23.78	9.63	8.75	18.38	5.38	23.75	60.44	9.57	40.29	52.18	14.64	66.82
149	Wc-1	N1589	Germany	52º36'	10º04' E	*fri*-Col	32.73	5.55	11.36	16.67	5.83	22.50	22.00	3.27	10.82	14.09	5.91	20.00	24.36	6.91	8.64	15.55	4.82	20.36	41.67	4.82	24.27	29.09	10.36	39.45
150	Wei-0	N6182	Switzerland	47º25'	8º26' E	*fri*-Ler	25.50	6.20	5.80	11.70	3.00	14.70	16.50	3.33	6.08	9.42	3.00	12.42	18.67	6.00	3.92	9.92	2.67	12.58	38.58	7.00	18.45	26.50	8.50	35.00
151	Wil-1	N1595	Lithuania	54º41	25º19' E	*FRI* non-functional	33.13	11.14	6.71	16.63	4.63	21.25	20.09	4.91	6.00	10.91	3.64	14.55	25.13	8.11	7.22	15.33	5.22	20.56	51.50	8.67	26.00	35.71	9.43	45.14
152	Wl-0	N1631	Germany	48º45'	8º33' E	*fri*-Ler	42.22	14.63	16.63	29.50	7.00	36.50	21.67	6.75	11.67	18.42	4.50	22.92	27.00	13.09	7.82	20.91	5.27	26.18	43.50	10.90	30.20	41.67	8.83	50.50
153	Wt-4	N1611	Germany	52º40'	9º48' E	*fri*-Col	32.00	9.38	10.38	20.00	5.50	25.50	20.73	5.82	9.82	15.64	5.27	20.91	27.91	9.91	12.27	22.18	5.82	28.00	37.50	8.75	17.75	26.50	9.00	35.50
154	Wü-0	N1615	Germany	49º47'47"	9º57'15' E	*fri*-Ler	28.27	7.50	7.30	14.82	4.45	19.27	19.67	3.75	9.00	12.75	4.83	17.58	22.00	8.18	7.55	15.73	4.73	20.45	51.88	9.50	41.00	50.50	11.60	62.10
155	Zü-0	N1627	Switzerland	47º25'	8º40' E		58.30	NA	NA	73.83	20.17	94.00	45.11	7.00	62.67	61.42	13.08	78.08	29.50	12.17	19.17	31.33	7.83	39.17	81.00	NA	NA	82.80	17.40	100.20
156	Col-0_A	Lab				*fri*-Col	31.50	9.00	7.71	16.60	3.70	20.30	20.08	4.58	7.75	12.33	3.17	15.50	21.83	8.00	5.58	13.58	3.58	17.17	56.40	NA	NA	49.17	9.17	58.33

157	Col-0_B	N1093		*fri*-Col	32.18	8.11	9.00	17.64	4.00	21.64	20.18	5.00	7.50	12.50	3.20	15.70	24.20	9.00	7.44	16.44	4.11	20.56	60.73	8.20	43.20	53.33	10.33	63.67
158	Col-0_C	Lab		*fri*-Col	25.42	7.33	9.67	17.00	4.08	21.08	20.78	4.78	7.11	11.89	3.33	15.22	21.25	6.60	6.40	13.00	3.60	16.60	NA	NA	NA	NA	NA	NA
159	Col-0_D	Lab		*fri*-Col	31.80	8.50	13.67	21.50	5.60	27.10	20.55	5.18	8.55	13.73	2.91	16.64	25.10	9.33	9.11	18.44	4.67	23.11	63.45	8.00	49.33	57.67	7.92	65.58
160	Ler_A	Lab		L*er*	26.50	8.13	3.13	11.45	3.09	14.55	16.91	3.64	5.00	8.64	3.09	11.73	22.18	8.00	4.27	12.27	3.91	16.18	51.14	NA	NA	27.71	8.14	35.86
161	Ler_B	Lab		L*er*	22.17	7.75	3.33	11.08	2.75	13.83	20.43	3.86	3.43	7.17	2.43	9.71	19.30	5.89	4.56	10.44	3.78	14.22	NA	NA	NA	NA	NA	NA
162	Ler_C	N6928		L*er*	26.91	8.14	2.86	11.50	3.00	14.50	17.40	4.00	4.44	8.44	3.44	11.89	22.00	7.10	5.20	12.30	3.70	16.00	53.22	8.25	18.25	28.25	8.38	36.63
			AVG		39.04	8.72	20.09	29.13	6.89	36.02	28.81	4.70	20.06	25.12	5.87	31.05	23.90	8.12	8.39	16.43	4.52	20.94	55.65	8.65	29.50	48.82	10.21	59.07
			SD		11.16	2.63	17.72	17.58	3.64	20.71	12.56	1.40	18.41	16.98	3.22	20.19	3.77	2.16	4.09	5.19	1.47	6.45	13.20	2.15	11.03	19.05	3.00	21.04

Mutants and Parents				16ºC Long Day (16LD)						23ºC Long Day (23LD)						16ºC Long Day + Vernalization (16LDV)						23ºC Short Day (23SD)					
No.	Genotype	Stock No	Background	DTF	JLN	ALN	RLN	CLN	TLN	DTF	JLN	ALN	RLN	CLN	TLN	DTF	JLN	ALN	RLN	CLN	TLN	DTF	JLN	ALN	RLN	CLN	TLN
1	Col-0	N1093	Col	30.40	8.73	8.36	17.09	3.45	20.55	18.43	4.25	7.88	12.13	3.00	15.13	22.60	6.40	4.80	11.20	3.40	14.60	57.33	8.67	40.89	49.56	9.44	59.00
2	Ler	N6928	Ler	30.33	9.67	2.83	12.50	4.17	16.67	17.50	4.20	6.80	11.00	3.20	14.20	22.00	7.14	2.86	10.00	3.71	13.71	58.70	7.75	21.00	28.30	10.70	39.00
3	ap1-15	Lab Stocl	Col	28.33	6.86	6.29	12.50	2.83	15.33	17.27	4.27	6.27	10.42	2.58	13.00	23.80	8.60	6.80	15.40	3.60	19.00	52.80	9.50	26.50	34.67	8.00	42.67
4	co-1	N3122	Ler	42.17	9.42	26.83	36.25	10.50	46.75	23.92	4.25	15.08	19.33	5.50	24.83	31.75	6.57	14.57	20.88	8.88	29.75	47.58	7.67	20.67	28.33	10.75	39.08
5	co-2	Lab Stock	Ler	45.30	9.00	22.00	27.60	9.60	37.20	26.33	4.25	14.00	18.33	5.11	23.44	32.50	7.67	16.33	24.40	10.00	34.40	47.43	8.60	18.40	26.30	8.90	35.20
6	cry2-1	N3732	Col	53.45	12.33	28.00	42.17	7.67	49.83	25.08	5.27	13.64	18.91	3.91	22.82	30.91	9.36	8.27	17.50	4.58	22.08	63.82	11.00	46.00	57.50	12.63	70.13
7	fca-1	N52	Ler	37.42	11.17	13.17	24.33	7.50	31.83	23.70	3.89	13.33	17.22	4.00	21.22	22.55	5.67	4.33	10.00	4.22	14.22	66.00	6.00	28.00	42.25	9.63	51.88
8	fca-2	N168	Ler	36.67	10.17	9.75	19.92	6.58	26.50	19.08	4.67	8.92	13.58	3.58	17.17	22.00	6.20	4.40	10.60	4.30	14.80	57.58	8.33	20.67	28.88	10.38	39.25
9	fca-3	N169	Ler	34.50	8.00	9.83	17.83	5.50	23.33	21.58	4.33	10.08	14.42	3.83	18.25	22.60	6.00	3.20	9.20	3.20	12.40	63.45	7.38	30.38	42.55	12.73	55.27
10	fca-4	N170	Ler	38.75	10.25	11.92	22.17	6.75	28.92	23.50	4.08	11.42	15.50	3.42	18.92	22.00	6.33	3.33	9.67	3.42	13.08	59.18	9.20	26.60	38.70	10.44	49.00
11	fca-5	N171	Ler	45.82	10.33	17.50	27.83	7.50	35.33	32.91	5.10	15.30	20.00	5.20	25.20	24.17	6.25	3.33	9.42	3.58	13.00	68.50	7.00	36.00	51.00	12.09	63.09
12	fca-6	N172	Ler	41.92	11.25	20.08	31.33	8.58	39.92	29.33	4.75	19.42	24.17	5.33	29.50	22.50	7.33	3.25	10.58	4.42	15.00	68.17	10.00	40.50	59.08	11.50	70.58
13	fd-1	Lab Stock	Ler	38.27	10.00	9.22	19.20	5.90	25.10	20.92	4.73	8.09	12.82	3.64	16.45	27.78	7.83	7.00	14.88	5.13	20.00	52.27	9.57	16.86	25.91	9.09	35.00
14	fe-1	N54	Ler	40.27	9.00	13.30	22.36	7.18	29.64	22.67	4.83	11.08	15.92	4.25	20.17	26.00	7.60	6.50	14.18	5.36	19.55	49.67	8.45	18.27	26.50	10.17	36.67
15	fha-1	N108	Ler	44.58	10.36	13.36	23.33	6.75	30.08	19.50	4.00	7.50	11.50	2.83	14.33	26.50	7.58	6.08	13.67	4.42	18.08	60.36	8.00	27.50	34.20	9.90	44.10
16	fha-2	N186	Ler	37.00	8.58	8.42	17.00	5.33	22.33	18.67	3.00	6.50	9.50	3.58	13.08	23.50	5.92	6.25	12.17	5.25	17.42	54.33	7.08	21.00	28.08	11.17	39.25
17	fha-3_A	N187	Ler	33.18	9.45	8.45	17.91	5.82	23.73	17.92	3.27	6.09	9.36	3.27	12.64	23.33	7.40	5.10	12.50	5.30	17.80	51.08	7.30	17.80	25.58	11.17	36.75
18	fha-3_B	Lab Stock	Ler	37.89	8.29	6.57	14.44	4.78	19.22	19.64	2.91	6.73	9.64	3.27	12.91	26.55	6.45	6.64	13.09	4.91	18.27	50.89	6.83	21.00	29.00	11.75	40.75
19	flc-3	Lab Stock	Col	31.18	7.64	6.36	14.00	2.91	16.91	17.83	4.08	6.75	10.83	2.75	13.58	22.00	7.30	5.70	13.00	3.80	16.80	56.50	8.00	40.09	48.09	9.73	57.82
20	fpa-1	N109	Ler	35.00	10.55	9.82	20.36	4.27	24.64	24.08	5.64	10.55	16.00	3.67	19.67	22.00	7.43	5.29	12.71	4.29	17.00	56.33	9.57	22.57	32.14	9.14	41.29
21	ft-1	Lab Stock	Ler	NA	8.89	12.00	20.90	8.10	29.00	28.00	5.67	10.92	16.58	5.83	22.42	28.78	8.80	9.60	18.40	7.20	25.60	NA	NA	NA	29.57	11.43	41.00
22	fve-1	N107	Ler	39.92	10.33	16.42	26.75	7.25	34.00	23.36	4.73	12.91	17.64	3.82	21.45	22.25	7.18	3.82	11.00	4.18	15.18	63.42	9.88	37.38	45.82	11.18	57.00
23	fwa-1	N106	Ler	41.67	8.70	12.80	21.50	8.92	30.42	23.73	5.09	11.36	16.45	5.27	21.73	28.86	7.17	8.33	14.63	7.88	22.50	58.75	7.80	21.40	29.58	12.33	41.92
24	fwa-2	Lab Stock	Ler	30.33	7.92	10.33	18.25	8.00	26.25	24.17	4.00	9.67	13.67	6.75	20.42	27.82	6.73	9.18	15.91	7.82	23.73	NA	NA	NA	28.00	11.75	39.75
25	fy-1_A	N57	Ler	39.75	9.00	11.83	20.83	5.25	26.08	24.73	4.09	10.82	14.91	3.73	18.64	24.25	6.00	4.45	10.58	3.33	13.92	63.17	8.00	31.00	37.70	9.00	46.70
26	fy-1_B	Lab Stock	Ler	35.08	8.00	13.50	21.50	6.17	27.67	26.08	5.17	9.08	16.75	3.92	18.17	24.42	6.67	5.50	12.17	4.25	16.42	NA	NA	NA	39.80	8.40	48.20
27	fy-1_C	Lab Stock	Ler	39.45	9.00	10.71	19.22	5.00	24.22	26.90	4.78	10.56	15.55	3.82	19.36	25.75	7.08	4.75	11.83	3.75	15.58	64.25	NA	NA	35.09	7.82	42.91
28	gi-6	N183	Ler	46.09	10.70	19.10	29.55	8.91	38.45	28.45	4.42	13.42	17.83	4.92	22.75	31.70	8.00	14.00	18.75	6.75	25.50	55.33	9.25	19.13	28.33	9.25	37.58
29	hy4-1 (cry1)	N70	Ler	31.50	9.75	7.83	17.58	5.92	23.50	19.00	4.00	7.83	11.83	3.92	15.75	24.83	7.09	5.45	12.55	5.00	17.55	60.58	9.67	41.22	52.00	13.73	64.82
30	ld-1	Lab Stock	Col	58.38	NA	NA	54.00	12.25	66.25	47.00	NA	NA	48.67	9.78	58.44	27.60	9.10	9.50	18.60	4.40	23.00	NA	NA	NA	NA	NA	NA
31	spy-1	Lab Stock	Ler	28.88	5.00	4.75	9.63	2.63	12.25	18.90	3.33	5.33	8.67	2.56	11.22	23.22	5.30	4.90	10.20	3.00	13.20	50.63	7.40	27.20	37.14	4.00	41.14
32	spy-2	Lab Stock	Ler	32.00	10.00	9.38	18.40	4.10	22.50	19.27	4.90	7.30	12.20	3.30	15.50	24.00	8.58	5.75	14.33	3.50	17.83	54.00	9.17	30.83	45.13	7.75	52.88
33	spy-3	Lab Stock	Ler	29.86	4.40	5.80	10.83	2.83	13.67	18.38	3.56	5.22	8.78	2.78	11.56	22.83	5.09	4.82	9.91	2.73	12.64	52.50	7.83	29.17	42.50	4.50	47.00
34	spy-5	Lab Stock	Ler	25.75	3.86	2.29	7.38	3.38	10.75	16.25	2.58	3.75	6.33	3.17	9.50	18.42	3.92	2.33	6.25	3.25	9.50	33.30	4.11	10.56	14.67	5.00	19.67
35	tfl1-1	Lab Stock	Col	22.92	NA	NA	9.10	0.80	9.90	15.80	NA	NA	9.11	1.22	10.33	16.60	NA	NA	10.90	1.33	12.10	49.00	NA	NA	44.43	7.29	51.71
36	toc1-1	Lab Stock	Ler	25.25	7.25	1.75	8.38	2.25	10.63	16.63	4.00	4.00	8.00	2.11	10.11	18.13	5.22	2.89	8.11	2.22	10.33	24.73	5.73	3.09	8.82	2.64	11.45
		AVG		36.84	8.94	11.49	20.94	5.98	26.93	22.68	4.30	9.64	14.82	3.97	18.72	24.62	6.94	6.27	13.03	4.62	17.65	55.36	8.16	26.39	35.86	9.58	45.41
		SD		7.73	1.87	6.27	9.46	2.54	11.71	5.90	0.74	3.60	7.06	1.50	8.37	3.74	1.19	3.31	3.80	1.84	5.39	9.16	1.44	9.86	11.25	2.56	12.37
