# Supplementary material for: Diversity of Flowering Responses in Wild Arabidopsis thaliana Strains
Source: PLoS Genet. 2005 Jul 25;1(1):e6. doi: 10.1371/journal.pgen.0010006 (PMC1183525; doi:10.1371/journal.pgen.0010006)
Supplement: Table S2 — Asterisk indicates ± 95% confidence intervals (2 × SEM). (31 KB PDF) [file pgen.0010006.st002.pdf]

Supplementary Table 2. Summary statistics based on individual plants.

Condition	Parameters	DTF	TLN
16LD	Mean*	38.5±0.6	34.2±1.1
	Standard deviation	12.18	20.78
	Median	36	26
	CV_g	28.12	56.94
	Range	20-86	8-114
	Sample size	1489	1377
	Heritability	0.81	0.9
23LD	Mean	28.0±0.6	29.6±1.0
	Standard deviation	13.0	20.2
	Median	23	20
	CV_g	43.11	64.45
	Range	14-82	7-136
	Sample size	1653	1643
	Heritability	0.85	0.89
16LDV	Mean	23.8±0.2	20.8±0.3
	Standard deviation	4.45	7.05
	Median	23	20
	CV_g	15.36	30.43
	Range	13-55	8-70
	Sample size	1679	1648
	Heritability	0.70	0.84
23SD	Mean	54.3±1.0	57.2±1.1
	Standard deviation	15.03	17.85
	Median	52	53
	CV_g	23.01	35.17
	Range	22-117	12-148
	Sample size	1187	1410
	Heritability	0.71	0.87

* ± 95% confidence intervals (2 x s.e.m.)
