# Supplementary material for: Diversity of Flowering Responses in Wild Arabidopsis thaliana Strains
Source: PLoS Genet. 2005 Jul 25;1(1):e6. doi: 10.1371/journal.pgen.0010006 (PMC1183525; doi:10.1371/journal.pgen.0010006)
Supplement: Table S3 — (33 KB PDF) [file pgen.0010006.st003.pdf]

**Supplementary Table 3.** Key to ATH1 array data.

| Accession | ATGE array IDs | Replicates |
|---|---|---|
| Bay-0 | ATGE_111_A,B,C | 3 |
| C24 | ATGE_112_A,C,D | 3 |
| Col-0 | ATGE_113_A,C | 2 |
| Cvi | ATGE_114_A,B,C | 3 |
| Est-1 | ATGE_115_A,B | 2 |
| Kin-0 | ATGE_116_A,B,C | 3 |
| Ler | ATGE_117_B,C | 2 |
| Nd-1 | ATGE_118_A,B,C | 3 |
| Shahdara | ATGE_119_A,C,D | 3 |
| Van-0 | ATGE_120_A,B,C | 3 |
| Ak-1 | ATGE_121_A | 1 |
| Bla-5 | ATGE_124_A | 1 |
| Can-0 | ATGE_125_A | 1 |
| Cen-0 | ATGE_126_A | 1 |
| CIBC10 | ATGE_127_A | 1 |
| Dra-1 | ATGE_128_A | 1 |
| Enk-T | ATGE_129_A | 1 |
| Er-0 | ATGE_130_A | 1 |
| Fr-2 | ATGE_131_A | 1 |
| GOT1 | ATGE_132_A | 1 |
| HR-5 | ATGE_133_A | 1 |
| Is-0 | ATGE_134_A | 1 |
| Li2:1 | ATGE_136_A | 1 |
| Nc-1 | ATGE_137_A | 1 |
| NFE1 | ATGE_138_A | 1 |
| Nok-1 | ATGE_139_A | 1 |
| Nw-1 | ATGE_140_A | 1 |
| M7323S | ATGE_141_A | 1 |
| Ms-0 | ATGE_142_A | 1 |
| Old-2 | ATGE_144_A | 1 |
| Ove-1 | ATGE_145_A | 1 |
| Se-0 | ATGE_146_A | 1 |
| Sf-2 | ATGE_147_A | 1 |
| Ta-0 | ATGE_148_A | 1 |
| Uk-3 | ATGE_149_A | 1 |
