# Supplementary material for: Diversity of Flowering Responses in Wild Arabidopsis thaliana Strains
Source: PLoS Genet. 2005 Jul 25;1(1):e6. doi: 10.1371/journal.pgen.0010006 (PMC1183525; doi:10.1371/journal.pgen.0010006)
Supplement: Table S4 — (33 KB PDF) [file pgen.0010006.st004.pdf]

Supplementary Table 4. Pearson correlation of known flowering regulators with Days to Flowering (DTF) and Total Leaf Number (TLN) in 23LD.

**Gene**	**ID**	**DTF**	**TLN**
FLC	At5g10140	0.7833	0.6397
ELF8	At2g06210	0.3964	0.3100
FRI	At4g00650	0.3826	0.4192
SPL9	At2g42200	0.3491	0.2995
TOE2	At5g60120	0.3349	0.1758
SPL3	At2g33810	0.2635	0.2381
SNZ	At2g39250	0.2464	0.1375
SMZ	At3g54990	0.2369	0.2099
EMF1	At5g11530	0.2218	0.1982
TOC1	At4g18020	0.2107	0.1278
SPL2	At5g43270	0.2076	0.1465
SPL6	At1g69170	0.1984	0.1453
EBS	At4g22140	0.1980	0.1609
GI	At1g22770	0.1795	0.1033
LHY	At1g01060	0.1357	0.2333
FUL	At5g60910	0.1259	0.1626
ELF6	At5g04240	0.1247	-0.0091
VIP2	At5g59710	0.1219	0.0798
FVE	At2g19520	0.1120	-0.0160
ELF4	At2g40080	0.1038	0.1106
FPF1	At5g24860	0.1020	0.1990
VIP3	At4g29830	0.0992	0.2011
CCA1	At2g46830	0.0947	0.2164
FCA	At4g16280	0.0779	0.0856
TFL1	At5g03840	0.0643	0.1812
FD	At4g35900	0.0530	0.1000
TOE1	At2g28550	0.0427	0.1105
FRS6	At1g52520	0.0375	0.0827
SPL4	At1g53160	0.0313	0.0681
ELF5	At5g62640	0.0220	-0.0227
PHYB	At2g18790	0.0209	0.0641
FRL1	At5g16320	0.0183	-0.0094
PHYA	At1g09570	0.0071	-0.0330
PFT1	AT1G25540	0.0023	0.0517
FRS9	At4g38170	0.0006	-0.0385
MAF5	At5g65080	-0.0013	-0.0570
SPL5	At3g15270	-0.0182	0.0649

FKF1	At1g68050	-0.0217	-0.0746
TFL2	At5g17690	-0.0265	-0.1365
FY	At5g13480	-0.0270	0.0008
FLM	At1g77070	-0.0353	0.0120
VRN2	At4g16845	-0.0431	-0.1480
EMF2	At5g51230	-0.0574	-0.0388
FLD	At3g10390	-0.0714	-0.1506
MAF2	At5g65050	-0.0747	-0.0570
SPL10	At1g27370	-0.0752	-0.1330
FT	At1g65480	-0.0793	-0.0118
CRY1	At4g08920	-0.0914	-0.1559
VIP4	At5g61150	-0.1064	-0.0367
FLM	At1g77080	-0.1220	-0.0537
VRN1	At3g18990	-0.1238	-0.1935
SPL11	At1g27360	-0.1290	-0.1075
FLK	At3g04610	-0.1505	-0.0876
LFY	At5g61850	-0.1539	-0.0836
HEN1	At4g20910	-0.1596	-0.3115
ELF7	At1g79730	-0.1861	-0.2738
CO	At5g15840	-0.1901	-0.1453
AGL24	At4g24540	-0.1921	-0.0696
ZTL	At5g57360	-0.1984	-0.1156
HUA2	At5g23150	-0.2085	-0.1597
MAF4	At5g65070	-0.2233	-0.1262
SPL13	At5g50570	-0.2465	-0.1668
FRL2	At1g31814	-0.2681	-0.1691
CRY2	At1g04400	-0.2843	-0.3587
SVP	At2g22540	-0.3355	-0.2931
ELF3	At2g25930	-0.3644	-0.3524
SOC1	At2g45660	-0.4101	-0.3901
SPL15	At3g57920	-0.4887	-0.4463
