# Supplementary material for: Diversity of Flowering Responses in Wild Arabidopsis thaliana Strains
Source: PLoS Genet. 2005 Jul 25;1(1):e6. doi: 10.1371/journal.pgen.0010006 (PMC1183525; doi:10.1371/journal.pgen.0010006)
Supplement: Table S5 — Uses: 1/3, amplification of 3.3-kb FRI genomic region; 1–13, FRI sequence analysis; 12/13, PCR genotyping of Col-type deletion in FRI; 3/4, PCR genotyping of Ler-type deletion in FRI; 6/7, FRI qRT-PCR; 30/31 and 28/34, amplification of 4.2-kb FLC genomic region (divided into two fragments); 14–35, FLC sequence analysis; 34/35, FLC cDNA amplification; 36–38, PCR genotyping of Ler-type insertion in FLC; 39/40, UBQ10 qRT-PCR; 41/42, FLC qRT-PCR. (36 KB PDF) [file pgen.0010006.st005.pdf]

Supplementary Table 5. Oligonucleotide primers used.

No	Lab designation	Sequence
1	CLFRI16	GTG AGT GTA TCT AGT GTT CA
2	CLFRI3	GTG GAA ATT AGG GCT TCT G
3	FRIP F	AGT ACT CAC AAG TCA CAA C
4	FRIP-R	GAA GAT CAT CGA ATT GGC
5	JW100	ATG TTA TTG CTT CAG AGG TT
6	JW159	AGG GCG TAG AGC ATT TAC
7	JW160	TAA TCC AAC TCT CAA TCT TCA
8	JW21	CGT ATG ACT TAG CTG TTG GAT
9	JW22	CTT GTG AGT CTC CAT ACA CTG
10	JW98	AAC GAC TTT TTC CTT TGA G
11	N-1078	GGA TAA CTA TTT GGG GTC TAA TGA TGA GTT ACT GCG
12	UJ26	AGA TTT GCT GGA TTT GAT AAG G
13	UJ34	ATA TTT GAT GTG CTC TCC
14	CT05	GTT ACT CAG TTA CTC TTT TTG
15	CT12	GGA TTT CAT TAT TTC CTT GG
16	CT13	CTT TGA ATC ACA ATC GTC GT
17	CT14	TG TAT CTT GTG TCT TTT GTC
18	CT15	ATT TTG ACA CGA GAT TAC TAA
19	CT17	ATG TAA AAG GTA AGG TGT TC
20	G-2513	GGA AGA ACA ATG TCG TGA AGA A
21	G-2526	GCT AGT ATT GAT GAC CCA TAA GAT
22	G-2527	TCC CTC AAA GAA AAG TCA TAC A
23	JA01	CAA ATC GTG AAT GAC ATG C
24	JA02	AAT TAG ACC AGT TTA TGT ACA GCA
25	JA03	GGC ACC AAA GAA ACA AGG CT
26	JA04	TAT CAG TCC TAT TGT GAA GTT AAG
27	JA05	ATA ATG ATG ATG TGG CGG TAA
28	JA06	TGT GAA TCT ATG TTG AAA TAA TTG AT
29	JA07	AGG ATC AAA ACT ACT AGC TAA CCC
30	JA08	CCT CCA GTT GAA CAA GAG CAT C
31	JA11	AGA TTG GGG CTG CGT TTA CAT TTT AT
32	JA15	ATT GAT TCA TAT TTT TCA TAC ACA G
33	JW177	TGT ATT TGG AGT TTG GCT TC
34	YK13	CGA GAA AAG GAA AAA AAA AAA TAG AAA GAG
35	YK14	CTC ACA CGA ATA AGG TAC AAA GTT CAT C
36	JW178	ACA TTG AGA GAA CAC CTT AC
37	JW179	TGG TCA CTT TTT TGG TTG CT

38	JW183	`ATT GGG GCT GCG TTT ACA`
39	G-1586	`TAA AAA CTT TCT CTC AAT TCT CTC T`
40	G-1587	`TTG TCG ATG GTG TCG GAG CTT`
41	G-1981	`AGA CAA GAA GAC CGA ACT CA`
42	G-1982	`TTT GTC CAG CAG GTG ACA TC`

Uses:

1/3: Amplification of 3.3 kb *FRI* genomic region

1-13: *FRI* sequence analysis

12/13: PCR genotyping of Col-type deletion in *FRI*

3/4: PCR genotyping of L*er*-type deletion in *FRI*

6/7: *FRI* qRT-PCR

30/31 and 28/34: Amplification of 4.2 kb *FLC* genomic region (divided into two fragments)

14-35: *FLC* sequence analysis

34/35: Amplification of *FLC* cDNA amplification

36-38: PCR genotyping of L*er*-type insertion in *FLC*

39/40: *UBQ10* qRT-PCR

41/42: *FLC* qRT-PCR
